# Effect of wear on the dynamic characteristics of a rigid rotor supported by journal bearings

**DOI:** 10.1038/s41598-024-60488-7

**Published:** 2024-04-30

**Authors:** Logamurthi Raja Moorthi, Jawaid Iqbal Inayat-Hussain, Azrul Abidin Zakaria

**Affiliations:** 1 College of Graduate Studies, Universiti Tenaga Nasional, Jalan IKRAM-UNITEN, 43000 Kajang, Selangor Malaysia; 2 Asset Operations and Maintenance, TNB Genco Sdn Bhd, Jalan Bangsar, 59200 Bangsar, Kuala Lumpur Malaysia; 3 College of Engineering, Universiti Tenaga Nasional, Jalan IKRAM-UNITEN, 43000 Kajang, Selangor Malaysia; 4 Institute of Power Engineering, Universiti Tenaga Nasional, Jalan IKRAM-UNITEN, 43000 Kajang, Selangor Malaysia; 5 Institute of Sustainable Energy, Universiti Tenaga Nasional, Jalan IKRAM-UNITEN, 43000 Kajang, Selangor Malaysia

**Keywords:** Rigid rotor, Wear, Dynamic coefficients, Journal bearing, Engineering, Mechanical engineering

## Abstract

The purpose of this work is to examine the effects of wear severity on the static and dynamic characteristics of journal bearings, and on the vibration response of a rigid rotor supported by journal bearings. Numerical simulations using MATLAB was conducted for three different operating regimes, namely low loaded operating regime ($\epsilon _0 = 0.15$), moderately loaded operating regime ($\epsilon _0 = 0.45$) and highly loaded operating regime ($\epsilon _0 = 0.75$) with wear depth parameter ratio ($\delta$) varied from 0 to 0.5 at increments of 0.1. Numerical results showed that the vibration response of the rotor generally increases with the increase of the wear depth for all cases of low, moderately and highly loaded operating regimes of the bearings. For the values of parameters considered in this work, it was shown that the vibration response amplitude of the rotor in worn journal bearings may be six times larger compared to the response amplitude of the rotor in non-worn bearings.

## Introduction

Due to their superior long lasting and load carrying capacity, journal bearings are commonly utilized to support high-speed rotating machinery such as compressors and turbines^1^. However, the bearings are exposed to a gradual wear development amidst the rotating machine’s operation, particularly during starts and stops or when passing through the rotor’s critical speed^2^. As such, the bearing’s geometry gets altered, thereby altering its dynamic characteristics^3^. Wear is amongst the most frequent failure that affects the bearing clearance at first hand and causes alterations to the rotating machine dynamics^4^. In the presence of wear, the pressure distribution, the rotor’s equilibrium position, the bearing’s dynamic coefficients and the system’s response changes due to the alteration in oil film thickness^5^.

In rotor dynamics, the dynamics of hydrodynamic bearings are tradiotionally represented using stiffness and damping coefficients. Therefore, a linearization of hydrodynamic forces around the shaft’s equilibrium position is required^6^. For that purpose, it is important to determine the forces created by the fluid flow to obtain precise estimates of the dynamics of the journal bearings. These forces can then be used to obtain the linearized stiffness and damping coefficients of the bearing. Linearized stiffness and damping coefficients are extensively utilized for rotor-bearing system’s stability and response analyses. Pressure gradients are determined by a first order perturbation of the force and the linearized coefficients are estimated by employing numerical integration on the pressure gradients. Childs^7^ as well as Yamamoto and Ishida^8^ revealed a methodology to determine linearized coefficients for both the long bearing (L/D > 2) and short bearing (L/D $\le$ 0.5) approximations. Turaga et al.^9^ employed a finite element method that accounts for the roughness on the bearing’s surface to determine the bearing’s linear coefficients. Rao and Sawicki^10^ established a harmonic combination of the long and short bearings approximations (the inverse sum of the inverse of both pressures), initially proposed by Hirani et al.^11^ to determine linearized coefficients for finite length bearings.

Several researchers have studied the behaviour of worn bearings utilized in rotating machines. Mokhtar et al.^12^ experimentally examined the characteristic of wear in plain hydrodynamic journal bearings under repeated start and stop sequences. The wear was limited to the area where the shaft was known to slide on the bearing’s surface during the early phases of the starting position; no wear was sustained while stopping since the shaft nearly stopped rotating before it made contact with the bearing. Dufrane et al.^13^ examined the wear present in steam turbine bearings by taking measurements at predetermined time frames to determine the nature and extent of wear. For a more in-depth examination of the effect of wear on journal bearing, two wear geometry models were developed and utilized. Fillon et al.^14^ studied the thermodynamic performance of a worn plain journal bearing. Because of its propensity to enter the wear-induced footprint, the worn bearing offers intriguing benefits like temperature drops. Bouyer et al.^15^ presented an experimental data obtained on a journal lobed bearing subjected to numerous starts and stops. It was discovered that wear changes the properties of bearings, resulting in a decrease in temperature overall and an increase in maximum pressure. In their study, it is shown that, for the case investigated, while bearing characteristics is clearly affected by wear, the bearing still remains useable and safe.

Moreover, Machado et al.^16^ developed a mathematical representation for the journal bearing’s static analysis by utilizing the Reynolds equation. They acquired the pressure distribution in the bearing and examined the performance of dissimilar geometries in severe conditions, high speeds, and high-applied load. Chasalevris et al.^17^ investigated the effect of worn journal bearings on the system response and specified the eventual development of additional frequency components. It was observed that sub-and superharmonics are revealed in the continuous wavelet transform (CWT) of the rotor-bearing system response for worn bearings. In the following year, Chasalevris et al.^18^ designed and investigated an experimental rotor-bearing system with an elastic rotor mounted in worn journal bearings. The system is operated at run-up and run-down conditions and the response is analyzed with priority on passage through the first critical speed. When compared to the intact system, wear adds more sub-and super-harmonics to the response signal. Papanikolaou et al.^5^ investigated the effect of wear of a short journal bearing on the rotordynamic stiffness and damping coefficients. Machado et al.^2^ introduced a mathematical model to represent geometric discontinuities in journal bearing. They further presented a mathematical model that described the wear present in journal bearings and the effect of the wear on the dynamic response of the rotor-bearing system in frequency domain^19^. Jamil et al.^20^ investigated the impact of wear in journal bearings on the dynamic behaviour of rotor bearing system relying upon the derivation of dynamic stiffness and damping coefficients of worn journal bearing. Kim et al.^21^ presented a review that summarized the previous studies done on journal bearing induced nonlinear, rotordynamic forces, and responses. Viana et al.^6^ investigated the boundaries for linear approximation of the hydrodynamic forces present in lobed hydrodynamic bearings in highly loaded operating conditions.

Whenever there is a change in the clearance of the bearing due to wear, the characteristics such as the static equilibrium position and the linearized dynamic coefficient will change. This will affect the vibration response of the rotor supported by the worn bearings. The purpose of the present paper is therefore to examine the effect of wear on the vibration response of a rigid rotor supported by worn bearings. In this paper, the wear model proposed by Dufrane et al.^13^ is used in the mathematical model of a short journal bearing. The oil film forces acting on the rotor and the dynamic coefficients (stiffness and damping) are computed for both cases of bearing without wear and bearing with wear. The dynamic coefficients are computed for different wear depth, and these coefficients are then used to determine the vibration response of a rigid rotor.

## Mathematical treatment

### Wear model

**Figure 1:**
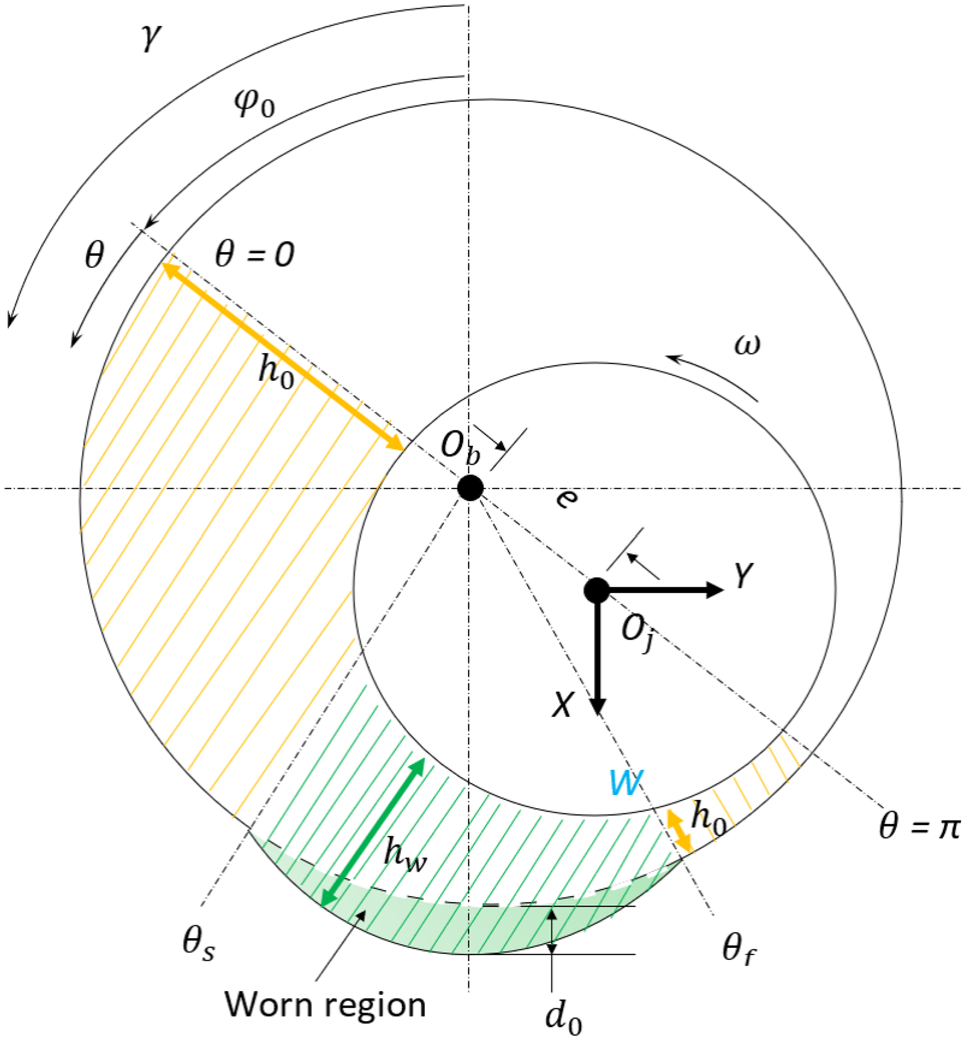
Wear model^5^.

The wear model used in this paper is the model proposed by Dufrane et al.^13^ and it is shown schematically in Fig. 1^5^. The region of positive pressure in the state of worn journal bearing can be divided into three sub regions^20^. The derivation of the equation presented herein follows the work of Papanikolaou et al.^5^ and Jamil et al.^20^.

#### $1^{st}$ Non-worn region $(0\le \theta \le \theta _s)$

In the $1^{st}$ non-worn region, the fluid-film thickness, $h_0$ , is expressed by Eq. (1).

1 $$\begin{aligned} h_0 = C + e_0cos\theta \end{aligned}$$

where,

$h_0$ is the fluid film thickness in the non-worn region (m).

C is the radial clearance (m).

$\epsilon _0$ is the eccentricity between the journal center and bearing center at equilibrium position (m).

$\theta$ is the angular coordinate measured from the position of maximum fluid-film thickness in the direction of rotor angular velocity (counter-clockwise), (rad).

For small amplitude motions about the journal equilibrium position, the fluid-film thickness, *h*, can be rewritten as shown in the following equation:

2 $$\begin{aligned} {h = C + e(t)cos\theta } \end{aligned}$$

where,

*h* is the fluid-film thickness for small amplitude motions about the journal equilibrium position (m).

$\theta =\gamma -\varphi _0$ ;$\gamma$ is the angle measured from the vertical axis, *X*, in the direction of rotor angular velocity (counter-clockwise), (rad).

$\varphi _0$ is the attitude angle measured from the vertical axis, *X*, to the position of maximum fluid-film thickness in the direction of rotor angular velocity (counter-clockwise), (rad).

The time-dependent eccentricity $\epsilon (t)$ and attitude angle $\varphi (t)$ are given by:

3 $$\begin{aligned} \begin{aligned}{}&e(t)= e_0+ \bigtriangleup {e}(t)\\&\varphi (t)= \varphi _0+ \bigtriangleup \varphi _0 \end{aligned} \end{aligned}$$

$\bigtriangleup {e}$ and $\bigtriangleup \varphi$ are small radial displacement and small variation of attitude angle respectively. Substituting Eq. (3) into Eq. (2) yields:

4 $$\begin{aligned} h=C+(e_0 + \bigtriangleup {e}){cos {\theta } cos{\bigtriangleup \varphi } + sin{\theta } sin{\bigtriangleup \varphi }} \end{aligned}$$

For small amplitude of motion, the following relationship is valid.

5 $$\begin{aligned} \begin{aligned}{}&cos\bigtriangleup \varphi \sim 1;\\&sin\bigtriangleup \varphi \sim \bigtriangleup \varphi ;\\&\bigtriangleup {e}\bigtriangleup \varphi = 0 \end{aligned} \end{aligned}$$

Substituting Eq. (5) into Eq. (4) gives the following expression for the instantaneous fluid-film thickness in the non-worn region.

6 $$\begin{aligned} h=C+e_0 cos{\theta } + \bigtriangleup {e}cos{\theta } + e_0{\bigtriangleup }{\varphi }sin{\theta } \end{aligned}$$

Differentiating Eq. (6) with respect to $\theta$ results in Eq. (7), which describes the change of fluid-film thickness in the circumferential direction of the bearing.

7 $$\begin{aligned} \frac{\partial {h}}{\partial {\theta }} = -e_0 sin{\theta } - {\bigtriangleup {e}}sin{\theta } + e_0{\bigtriangleup }{\varphi }cos{\theta } \end{aligned}$$

Differentiating Eq. (6) with respect to time, *t*, results in Eq. (8), which describes the change, with time, of the fluid-film thickness at a fixed point in the bearing.

8 $$\begin{aligned} \frac{\partial {h}}{\partial {t}} = \bigtriangleup {\dot{e}}cos{\theta } + e_0{\bigtriangleup {\dot{\varphi }}sin{\theta }} \end{aligned}$$

#### Worn region $(\theta _s\le \theta \le \theta _f)$

In the worn region, the fluid-film thickness, $h_{0w}$, is expressed by Eq. (9).

9 $$\begin{aligned} h_{0w} = d_0 + e_0 cos{\theta } -Ccos (\theta + \varphi _0) \end{aligned}$$

where,

$h_{0w}$ is the worn fluid film thickness at equilibrium position (m);

*C* is the journal bearing radial clearance (m).

$d_0$ is the wear depth (m).

Substituting Eq. (3) into Eq. (9) yields:

10 $$\begin{aligned} h_w = d_0 + (e_0 + \bigtriangleup {e})cos (\gamma - \varphi _0 - \bigtriangleup {\varphi })- Ccos (\theta + \varphi _0 ) \end{aligned}$$

Substituting Eq. (5) into Eq. (10) gives the following expression for the instantaneous fluid-film thickness in worn region.

11 $$\begin{aligned} h_w = d_0 + e_0cos{\theta } - Ccos(\theta + \varphi _0 ) + e_0{\bigtriangleup }{\varphi }sin{\theta } + \bigtriangleup {e}cos{\theta } \end{aligned}$$

Differentiating Eq. (11) with respect to $\theta$ yields Eq. (12), which describes the change of fluid-film thickness in the circumferential direction of the bearing.

12 $$\begin{aligned} \frac{\partial {h_w}}{\partial {\theta }} = Csin({\theta } + \varphi _0) - e_0sin{\theta } - \bigtriangleup {e_0}sin{\theta }+ e_0{\bigtriangleup {\varphi }}cos{\theta } \end{aligned}$$

Differentiating Eq. (11) with respect to time, *t*, yields Eq. (13), which describes the change, with time, of the fluid-film thickness at a fixed point in the bearing.

13 $$\begin{aligned} \frac{\partial {h_w}}{\partial {t}} = \bigtriangleup {\dot{e}}cos{\theta } + e_0{\bigtriangleup }{\dot{\varphi }}sin{\theta } \end{aligned}$$

#### $2^{nd}$ Non-worn region $(\theta _f\le \theta \le \pi )$

In this region the thickness of lubricant oil fluid is as in the $1^{st}$ non-worn region. The starting and final angles of the region where the wear takes place (refer to Fig. 1) are given by:

14 $$\begin{aligned} cos{(\theta + \varphi _0)} = \frac{d_0}{c} -1 = \delta - 1 \end{aligned}$$

where,

$$\delta = \frac{d_0}{c}$$;

$\delta$ is the wear depth parameter.

$\theta _s$ and $\theta _f$ are represented by the following equations^20^:

15 $$\begin{aligned} \theta _s= & {} \pi - \varphi _0 - arcos{(1-\delta )} \end{aligned}$$

16 $$\begin{aligned} \theta _f= & {} \pi - \varphi _0 + arcos{(1-\delta )} \end{aligned}$$

### Solution to bearing forces and dynamic coefficients

The fluid film pressure is obtained by solving the Reynolds equation for infinitely short bearing (L/D < 0.5). The bearing forces are computed by integrating the fluid film pressure over the bearing area using Simpson’s 1/3 rule.

#### Reynolds equation

For convenience purposes, polar coordinates is employed instead of Cartesian coordinates to solve the Reynolds equation. The polar coordinates used are the eccentricity ratio, $\epsilon _0$ and the attitude angle $\varphi _0$.

With the following assumptions: (i) short journal bearing, (ii) laminar fluid flow, (iii) iso-viscous fluid, (iv) incompressible fluid, and (v) $\pi$-film cavitation model, the Reynolds equation is written as follows^20^:

17 $$\begin{aligned} \frac{\partial }{\partial {Z}}{\left( \frac{h^3}{12\mu } \frac{\partial {P}}{\partial {Z}}\right) }= \frac{\partial {h}}{\partial {t}}+\frac{\Omega }{2} \frac{\partial {h}}{\partial {\theta }} \end{aligned}$$

where,

$\Omega$ is the rotational speed of the rotor (rad/s)

$\mu$ is the fluid viscosity (Pa.s)

By integrating the Reynolds equation with respect to *Z*, the pressure distribution for the non-worn and worn regions are obtained^20^:

Non-worn region: The pressure distribution in the non-worn region, *P*, is expressed by Eq. (18).

18 $$\begin{aligned} P(\theta ,Z,t) = \frac{6\mu }{h^3}\left( \frac{\partial {h}}{\partial {t}}+\frac{\Omega }{2} \frac{\partial {h}}{\partial {\theta }}\right) \left( Z^2 - \frac{L^2}{4}\right) \end{aligned}$$

where,

*L* is the journal bearing length (m).

*Z* is the axial distance in the direction of *Z*-axis

Worn region: The pressure distribution in the worn region, $P_w$, is expressed by Eq. (19).

19 $$\begin{aligned} P_w(\theta ,Z,t) = \frac{6\mu }{h^3}\left( \frac{\partial {h_w}}{\partial {t}}+\frac{\Omega }{2} \frac{\partial {h_w}}{\partial {\theta }}\right) \left( Z^2 - \frac{L^2}{4}\right) \end{aligned}$$

where,

$P_w$ is the pressure in the worn region of the journal bearing;

$h_w$ is the fluid film thickness in the worn region of the journal bearing.

#### Dynamic coefficients of worn journal bearings

The radial and tangential components of fluid film journal bearing forces are presented in Eq. (20)^20^:

20 $$\begin{aligned} \left\{ \begin{array}{l} F_r \\ F_t\end{array}\right\} = 2\int ^{L/2}_{0}\int ^{\pi }_{0} P(\theta , Z, t) R \left( \begin{array}{l} cos{\theta } \\ sin{\theta }\end{array}\right) d{\theta }d{z} \end{aligned}$$

Substituting the equation for pressure distribution in the non-worn regions and worn region of fluid film journal bearing into Eq. (20) gives:

21 $$\begin{aligned} \begin{aligned} \left\{ \begin{array}{l} F_r \\ F_t\end{array}\right\}&=\frac{12\mu }{h^3} R \int ^{\frac{L}{2}}_0 \left\{ \int ^{\theta _s}_{0} \left( \frac{\partial {h}}{\partial {t}}\right. \left. + \frac{\Omega }{2} \frac{\partial {h}}{\partial {\theta }}\right) + \int ^{\theta _f}_{\theta _s} \left( \frac{\partial h_w}{\partial t} + \frac{\Omega }{2} \frac{\partial h_w}{\partial \theta }\right) \right. \\&\quad \left. + \int ^{\pi }_{\theta _f} \left( \frac{\partial {h}}{\partial {t}}+\frac{\Omega }{2} \frac{\partial {h}}{\partial {\theta }}\right) \right\} \left. \left( \begin{array}{l} cos{\theta }\\ sin{\theta }\end{array}\right) \right. \left. \left( Z^2 - \frac{L^2}{4}\right) d{\theta }d{z}\right. \end{aligned} \end{aligned}$$

Substituting Eq. (21) with Eqs. (7), (8), (12) and (13) and integrating the result with respect to *Z* gives the following expression:

22 $$\begin{aligned} \begin{aligned} \left\{ \begin{array}{l} F_r \\ F_t\end{array}\right\} =&-\frac{\Omega }{2}\frac{\mu {L^3}R}{{c^3}{H^3}} \int ^{\theta _s}_0 \left\{ \begin{array}{l} {-e_0}sin{\theta }cos{\theta }\\ {-e_0}sin^2{\theta }\end{array}\right\} d{\theta } -\frac{\Omega }{2}\frac{\mu {L^3}R}{{c^3}{H^3}} \int ^{\pi }_{\theta _f} \left\{ \begin{array}{l} {-e_0}sin{\theta }cos{\theta } \\ {-e_0}sin^2{\theta }\end{array}\right\} d{\theta }\\&-\frac{\Omega }{2}\frac{\mu {L^3}R}{{c^3}{H_{w}^3}} \int ^{\theta _f}_{\theta _s} \left\{ \begin{array}{l} {-e_0}sin{\theta }cos{\theta }+csin({\theta +\varphi _0})cos{\theta } \\ {-e_0}sin^2{\theta }\end{array}\right\} d{\theta }\\&-\frac{\Omega }{2}\frac{\mu {L^3}R}{{c^3}{H^3}}\left[ \int ^{\theta _s}_0 \left\{ \begin{array}{l} -sin{\theta }cos{\theta }\\ -sin^2{\theta }\end{array}\right\} d{\theta }\right] {\bigtriangleup }e -\frac{\Omega }{2}\frac{\mu {L^3}R}{{c^3}{H^3}}\left[ \int ^{\theta _s}_0 \left\{ \begin{array}{l} cos^2{\theta }\\ sin{\theta }cos{\theta }\end{array}\right\} d{\theta }\right] e_0{\bigtriangleup }\varphi \\&-\frac{\Omega }{2}\frac{\mu {L^3}R}{{c^3}{H_w^3}}\left[ \int ^{\theta _f}_{\theta _s} \left\{ \begin{array}{l} -sin{\theta }cos{\theta }\\ -sin^2{\theta }\end{array}\right\} d{\theta }\right] {\bigtriangleup }e -\frac{\Omega }{2}\frac{\mu {L^3}R}{{c^3}{H^3}}\left[ \int ^{\theta _f}_{\theta _s} \left\{ \begin{array}{l} cos^2{\theta }\\ sin{\theta }cos{\theta }\end{array}\right\} d{\theta }\right] e_0{\bigtriangleup }\varphi \\&-\frac{\Omega }{2}\frac{\mu {L^3}R}{{c^3}{H^3}}\left[ \int ^{\pi }_{\theta _f} \left\{ \begin{array}{l} -sin{\theta }cos{\theta }\\ -sin^2{\theta }\end{array}\right\} d{\theta }\right] {\bigtriangleup }e -\frac{\Omega }{2}\frac{\mu {L^3}R}{{c^3}{H^3}}\left[ \int ^{\pi }_{\theta _f} \left\{ \begin{array}{l} cos^2{\theta }\\ sin{\theta }cos{\theta }\end{array}\right\} d{\theta }\right] e_0{\bigtriangleup }\varphi \\&-\frac{\Omega }{2}\frac{\mu {L^3}R}{{c^3}{H^3}}\left[ \int ^{\theta _s}_0 \left\{ \begin{array}{l} cos^2{\theta }\\ sin{\theta }cos{\theta }\end{array}\right\} d{\theta }\right] {\bigtriangleup }\dot{e} -\frac{\Omega }{2}\frac{\mu {L^3}R}{{c^3}{H^3}}\left[ \int ^{\theta _s}_0 \left\{ \begin{array}{l} sin{\theta }cos{\theta }\\ sin^2{\theta }\end{array}\right\} d{\theta }\right] e_0{\bigtriangleup }\dot{\varphi }\\&-\frac{\Omega }{2}\frac{\mu {L^3}R}{{c^3}{H_w^3}}\left[ \int ^{\theta _f}_{\theta _s} \left\{ \begin{array}{l} cos^2{\theta }\\ sin{\theta }cos{\theta }\end{array}\right\} d{\theta }\right] {\bigtriangleup }\dot{e} -\frac{\Omega }{2}\frac{\mu {L^3}R}{{c^3}{H_w^3}}\left[ \int ^{\theta _f}_{\theta _s} \left\{ \begin{array}{l} sin{\theta }cos{\theta }\\ sin^2{\theta }\end{array}\right\} d{\theta }\right] e_0{\bigtriangleup }\dot{\varphi }\\&-\frac{\Omega }{2}\frac{\mu {L^3}R}{{c^3}{H^3}}\left[ \int ^{\pi }_{\theta _f} \left\{ \begin{array}{l} cos^2{\theta }\\ sin{\theta }cos{\theta }\end{array}\right\} d{\theta }\right] {\bigtriangleup }\dot{e} -\frac{\Omega }{2}\frac{\mu {L^3}R}{{c^3}{H^3}}\left[ \int ^{\pi }_{\theta _f} \left\{ \begin{array}{l} sin{\theta }cos{\theta }\\ sin^2{\theta }\end{array}\right\} d{\theta }\right] e_0{\bigtriangleup }\dot{\varphi }\\ \end{aligned} \end{aligned}$$

where,

$$H=\frac{h}{c}$$ and $$H_w=\frac{h_w}{c}$$

The solution of Eq. (22) has the following form^22^:

23 $$\begin{aligned} \left[ \begin{array}{l} F_r \\ F_t\end{array}\right] = \left[ \begin{array}{l} F_{r0} \\ F_{t0}\end{array}'\right] - \left[ \begin{array}{cc} K_{rr} &{} K_{rt} \\ K_{tr} &{} K_{tt} \\ \end{array}\right] \left[ \begin{array}{cc} \bigtriangleup {e} \\ e_0\bigtriangleup \varphi \\ \end{array}\right] - \left[ \begin{array}{cc} C_{rr} &{} C_{rt} \\ C_{tr} &{} C_{tt} \\ \end{array}\right] \left[ \begin{array}{cc} \bigtriangleup {\dot{e}} \\ {e_0}{\bigtriangleup }\dot{\varphi } \\ \end{array}\right] \end{aligned}$$

Simpsons 1/3 rule is used to solve Eq. (23) and the solution can be described as:

24 $$\begin{aligned} \begin{aligned} F_{r0}&= c.{F_s}\left\{ \int ^{\theta _s}_{0} \frac{{\epsilon _0}sin{\theta }cos{\theta }}{H^3_0}d\theta + \int ^{\theta _f}_{\theta _s} \frac{{\epsilon _0}sin{\theta }cos{\theta }-cos{\theta }sin({\theta +\varphi _0})}{H^3_{0w}}d\theta \right. \\&\quad \left. +\int ^{\pi }_{\theta _f} \frac{{\epsilon _0}sin{\theta }cos{\theta }}{H^3_0}d\theta \right\} \\ F_{t0}&= c.{F_s}\left\{ \int ^{\theta _s}_{0} \frac{{\epsilon _0}(sin{\theta })^2}{H^3_0}d\theta + \int ^{\theta _f}_{\theta _s} \frac{{\epsilon _0}(sin{\theta })^2-sin{\theta }sin({\theta +\varphi _0})}{H^3_{0w}}d\theta \right. \\&\quad \left. +\int ^{\pi }_{\theta _f} \frac{{\epsilon _0}(sin{\theta })^2}{H^3_0}d\theta \right\} \\ K_{rr}&= {F_s}\left\{ -\int ^{\theta _s}_{0}\frac{sin{\theta }cos{\theta }}{H^3_0}d\theta + 3.{\epsilon _0}.\int ^{\theta _s}_{0} \frac{sin{\theta }(cos{\theta })^2}{H^4_0}d\theta \right. \\&\quad \left. - \int ^{\theta _f}_{\theta _s} \frac{sin{\theta }cos{\theta }}{H^3_{0w}}d\theta + 3.\epsilon _0.\int ^{\theta _f}_{\theta _s} \frac{sin{\theta }(cos{\theta })^2}{H^4_{0w}}d\theta \right. \\&\quad \left. - 3.\int ^{\theta _f}_{\theta _s} \frac{(cos{\theta })^2sin({\theta +\varphi _0})}{H^4_{0w}}d\theta -\int ^{\pi }_{\theta _f} \frac{sin{\theta }cos{\theta }}{H^3_{0}}d\theta \right. \\&\quad \left. + 3.\epsilon _0.\int ^{\pi }_{\theta _f} +\frac{sin{\theta }(cos{\theta })^2}{H^4_{0}}d\theta \right\} \\ K_{rt}&= {F_s}\left\{ \int ^{\theta _s}_{0}\frac{(cos\theta )^2}{H^3_0}d\theta + 3.\epsilon _0.\int ^{\theta _s}_{0} \frac{cos{\theta }(sin\theta )^2}{H^4_0}d\theta \right. \\&\quad \left. +3.\epsilon _0. \int ^{\theta _f}_{\theta _s} \frac{(sin\theta )^2cos\theta }{H^4_{0w}}d\theta + \int ^{\theta _f}_{\theta _s} \frac{(cos{\theta })^2}{H^3_{0w}}d\theta \right. \\&\quad \left. - 3.\int ^{\theta _f}_{\theta _s} \frac{cos{\theta }sin({\theta +\varphi _0})}{H^4_{0w}}d\theta +\int ^{\pi }_{\theta _f} \frac{(cos{\theta })^2}{H^3_{0}}d\theta \right. \\&\quad \left. + 3.\epsilon _0.\int ^{\pi }_{\theta _f} +\frac{cos{\theta }(sin{\theta })^2}{H^4_{0}}d\theta \right\} \\ K_{tr}&= {F_s}\left\{ -\int ^{\theta _s}_{0}\frac{(sin\theta )^2}{H^3_0}d\theta + 3.\epsilon _0.\int ^{\theta _s}_{0} \frac{cos{\theta }(sin\theta )^2}{H^4_0}d\theta \right. \\&\quad \left. +3.\epsilon _0. \int ^{\theta _f}_{\theta _s} \frac{(sin\theta )^2cos\theta }{H^4_{0w}}d\theta - \int ^{\theta _f}_{\theta _s} \frac{(sin{\theta })^2}{H^3_{0w}}d\theta \right. \\&\quad \left. - 3.\int ^{\theta _f}_{\theta _s} \frac{sin{\theta }cos{\theta }sin({\theta +\varphi _0})}{H^4_{0w}}d\theta -\int ^{\pi }_{\theta _f} \frac{(sin{\theta })^2}{H^3_{0}}d\theta \right. \\&\quad \left. + 3.\epsilon _0.\int ^{\pi }_{\theta _f} +\frac{cos{\theta }(sin{\theta })^2}{H^4_{0}}d\theta \right\} \\ K_{tt}&= {F_s}\left\{ \int ^{\theta _s}_{0}\frac{sin{\theta }cos{\theta }}{H^3_0}d\theta + 3.\epsilon _0.\int ^{\theta _s}_{0} \frac{(sin\theta )^3}{H^4_0}d\theta \right. \\&\quad \left. +3.\epsilon _0. \int ^{\theta _f}_{\theta _s} \frac{(sin\theta )^3}{H^4_{0w}}d\theta + \int ^{\theta _f}_{\theta _s} \frac{(sin{\theta }cos{\theta }}{H^3_{0w}}d\theta \right. \\&\quad \left. - 3.\int ^{\theta _f}_{\theta _s} \frac{{(sin{\theta })^2}sin({\theta +\varphi _0})}{H^4_{0w}}d\theta +\int ^{\pi }_{\theta _f} \frac{sin{\theta }cos{\theta }}{H^3_{0}}d\theta \right. \\&\quad \left. + 3.\epsilon _0.\int ^{\pi }_{\theta _f} +\frac{(sin{\theta })^3}{H^4_{0}}d\theta \right\} \\ C_{rr}&= \frac{2F_s}{\Omega }\left\{ \int ^{\theta _s}_{0}\frac{(cos\theta )^2}{H^3_0}d\theta +\int ^{\theta _f}_{\theta _s}\frac{(cos\theta )^2}{H^3_{0w}}d\theta +\int ^{\pi }_{\theta _f}\frac{(cos\theta )^2}{H^3_0}d\theta \right\} \\ C_{rt}&= \frac{2F_s}{\Omega }\left\{ \int ^{\theta _s}_{0}\frac{sin{\theta }cos{\theta }}{H^3_0}d\theta +\int ^{\theta _f}_{\theta _s}\frac{sin{\theta }cos{\theta }}{H^3_{0w}}d\theta +\int ^{\pi }_{\theta _f}\frac{sin{\theta }cos{\theta }}{H^3_0}d\theta \right\} \\ C_{tr}&= \frac{2F_s}{\Omega }\left\{ \int ^{\theta _s}_{0}\frac{sin{\theta }cos{\theta }}{H^3_0}d\theta +\int ^{\theta _f}_{\theta _s}\frac{sin{\theta }cos{\theta }}{H^3_{0w}}d\theta +\int ^{\pi }_{\theta _f}\frac{sin{\theta }cos{\theta }}{H^3_0}d\theta \right\} \\ C_{tt}&= \frac{2F_s}{\Omega }\left\{ \int ^{\theta _s}_{0}\frac{(sin\theta )^2}{H^3_0}d\theta +\int ^{\theta _f}_{\theta _s}\frac{(sin\theta )^2}{H^3_{0w}}d\theta +\int ^{\pi }_{\theta _f}\frac{(sin\theta )^2}{H^3_0}d\theta \right\} \end{aligned} \end{aligned}$$

where,

$$F_s = \frac{\mu \Omega R L^3}{2c^3}$$

The dynamic coefficients $K_{ij}$ and $C_{ij}$ refer to the rotating coordinate system and not to the Cartesian coordinate system. The relationship between the dynamic coefficients in both coordinate systems can be determined by the following transformations^5^:

25 $$\begin{aligned} \left[ \begin{array}{cc} K_{yx} &{} K_{xy} \\ K_{yx} &{} K_{yy} \\ \end{array}\right]= & {} \left[ \begin{array}{cc} cos{\varphi _0} &{} -sin{\varphi _0} \\ sin{\varphi _0} &{} cos{\varphi _0}\\ \end{array}\right] \left[ \begin{array}{cc} K_{rr} &{} K_{rt} \\ K_{tr} &{} K_{tt} \\ \end{array}\right] \left[ \begin{array}{cc} cos{\varphi _0} &{} sin{\varphi _0} \\ -sin{\varphi _0} &{} cos{\varphi _0} \\ \end{array}\right] \nonumber \\ \left[ \begin{array}{cc} C_{yx} &{} C_{xy} \\ C_{yx} &{} C_{yy} \\ \end{array}\right]= & {} \left[ \begin{array}{cc} cos{\varphi _0} &{} -sin{\varphi _0} \\ sin{\varphi _0} &{} cos{\varphi _0} \\ \end{array}\right] \left[ \begin{array}{cc} C_{rr} &{} C_{rt} \\ C_{tr} &{} C_{tt} \\ \end{array}\right] \left[ \begin{array}{cc} cos{\varphi _0} &{} sin{\varphi _0} \\ -sin{\varphi _0} &{} cos{\varphi _0} \\ \end{array}\right] \end{aligned}$$

For the worn journal bearing, the attitude angle $\varphi _0$ can be determined from Eq. (26)

26 $$\begin{aligned} tan^{-1}{\varphi _0} = -\frac{F_{t0}}{F_{r0}} \end{aligned}$$

However, in the case of non-worn bearing, $\varphi _0$ can be related to $\epsilon _0$, by the following expression^22^.

27 $$\begin{aligned} tan^{-1}{\varphi _0} = -\frac{\pi \sqrt{\left( 1-{\epsilon _{0}^2}\right) }}{4\epsilon _0} \end{aligned}$$

### Equation of motion of a rigid rotor in journal bearing

In the formulation of the equations of motion, the rotor is assumed to be rigid, symmetric and with a centrally-located mass. Therefore, only the lowest mode (cylindrical) can be excited by the applied unbalance. Rotor motion in the axial direction is neglected, and only the motion in the vertical (*X*) and horizontal (*Y*) directions are considered in the formulation of the equations of motion. Let the rotor mass be 2*M* and the journal center amplitudes $\bar{x}$ and $\bar{y}$. Hence, the equations of motion can be written^22^:

28 $$\begin{aligned} \begin{aligned}{}&M\frac{d^2{\bar{x}}}{dt^2} = F_x + MU\omega ^2cos{\omega {t}}\\&M\frac{d^2{\bar{y}}}{dt^2} = F_y + MU\omega ^2sin{\omega {t}} \end{aligned} \end{aligned}$$

Here $F_x$ and $F_y$ are the journal bearing film forces. The equations are made dimensionless by setting^23^:

29 $$\begin{aligned} \begin{aligned}{}&x = \frac{\bar{x}}{C}; y = \frac{\bar{y}}{C}; u = \frac{\bar{U}}{C}; \tau = \omega {t}\\&f_x = \frac{1}{\sigma } \frac{F_x}{W}\\&f_y = \frac{1}{\sigma } \frac{F_y}{W}\\&m = \frac{1}{\sigma } \frac{CM\omega ^2}{W}\\ \end{aligned} \end{aligned}$$

where modified Sommerfeld number:

30 $$\begin{aligned} \sigma = \frac{1}{8} \frac{\mu {\omega }DL}{W} \left( \frac{L}{C}\right) ^2 \end{aligned}$$

$\mu$ is the lubricant viscosity, $\omega$ is the angular speed, *D* is the bearing diameter, *L* is the bearing length, *W* is the static load on the bearing, *C* is the radial bearing clearance.

The modified Sommerfeld number, $\sigma$, can be related to the eccentricity ratio, $\epsilon _0$, by the following expression.

31 $$\begin{aligned} \sigma = \frac{(1-{\epsilon _0}^2)^2}{\epsilon _{0}\sqrt{16\epsilon _{0}^2 + \pi ^2(1-\epsilon _{0}^2)}} \end{aligned}$$

Letting “dot” refer to $d/d\tau$ and substituting Eq. (29) into Eq. (28), the dimensionless equations of motion become:

32 $$\begin{aligned} \begin{aligned}{}&m\ddot{x} = f_x + m{u}\omega ^2cos\tau \\&m\ddot{y} = f_y + m{u}\omega ^2sin\tau \end{aligned} \end{aligned}$$

where,

$$\begin{aligned} \begin{aligned}{}&{f_x = -K_{xx}{x} - K_{xy}{y} - C_{xx}{\dot{x}} - C_{xy}{\dot{y}}}\\&{f_y = -K_{yy}{y} - K_{yx}{x} - C_{yx}{\dot{x}} - C_{yy}{\dot{y}}} \end{aligned} \end{aligned}$$

Hence, Eq. (32) can be written in the following form:

33 $$\begin{aligned} \left[ \begin{array}{cc} m &{} 0 \\ 0 &{} m \\ \end{array}\right] \left[ \begin{array}{cc} {\ddot{x}} \\ {\ddot{y}} \\ \end{array}\right] + \left[ \begin{array}{cc} C_{xx} &{} C_{xy} \\ C_{yx} &{} C_{yy} \\ \end{array}\right] \left[ \begin{array}{cc} {\dot{x}} \\ {\dot{y}} \\ \end{array}\right] + \left[ \begin{array}{cc} K_{xx} &{} K_{xy} \\ K_{yx} &{} K_{yy} \\ \end{array}\right] \left[ \begin{array}{cc} {x} \\ {y} \\ \end{array}\right] = \left[ \begin{array}{cc} mu\omega ^2{cos\tau } \\ mu\omega ^2{sin\tau } \\ \end{array}\right] \end{aligned}$$

Eq. (33) is solved for *x* and *y* using MATLAB linear solver.

## Results and discussion

### Effect of wear on the static equilibrium position

**Figure 2:**
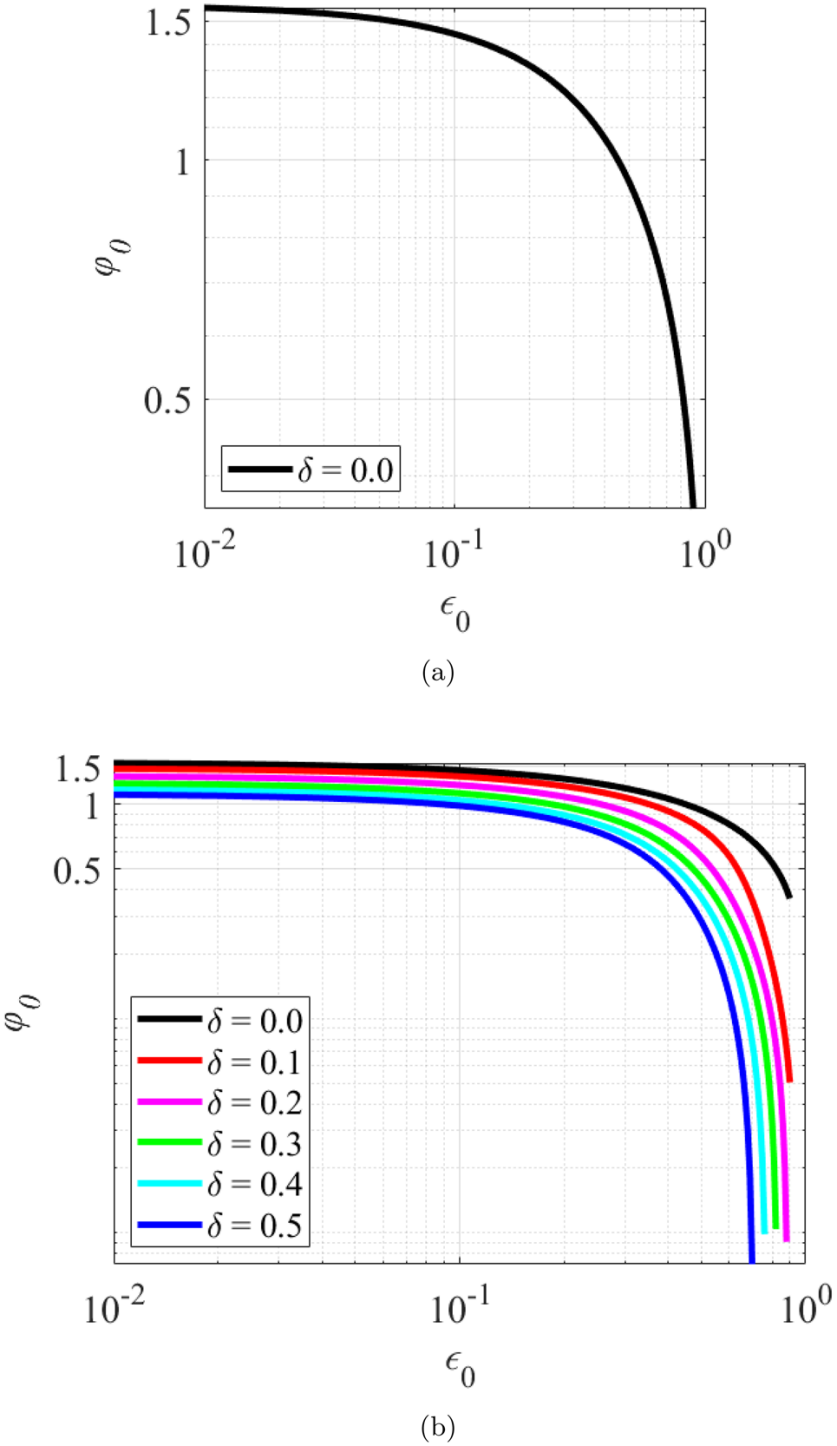
Relationship between $\epsilon _0$ and $\varphi _0$ for (**a**) bearing without wear (**b**) worn bearing.

The effect of wear on the static equilibrium position was examined for values of $\epsilon _0$ between 0.1 and 0.9. The wear depth parameter ratio $\delta$ was varied from 0 to 0.5, in increments of 0.1. Figure 2a shows the relationship between $\varphi _0$ and $\epsilon _0$ for bearing without wear. It is observed that $\epsilon _0$ has an inverse relationship with $\varphi _0$ whereby that $\epsilon _0$ increases as $\varphi _0$ decreases. To understand the relationship between $\varphi _0$ and $\epsilon _0$, the relationship between $\sigma$ and $\epsilon _0$ needs to be understood first as expressed in Eq. (31). Equation (31) provides a relationship to determine $\epsilon _0$ required to generate the fluid film reaction force. Large $\sigma$ represents low operating load, high $\omega$ or high fluid viscosity, $\mu$, which results in small $\epsilon _0$ or nearly centered operation, $\epsilon _0\rightarrow {0}$, $\varphi _0 \rightarrow \pi /2$. That is, the journal eccentricity vector is nearly orthogonal to the applied load. Small $\sigma$ represents high operating load, low $\omega$ or small $\mu$ , which results in large $\epsilon _0$, $\epsilon _0\rightarrow 1.0$, $\varphi _0\rightarrow 0$. That is, the journal eccentricity vector is nearly parallel to the applied load. The relationship between $\varphi _0$ and $\epsilon _0$ is expressed in Eq. (27) and this equation is only valid to calculate $\varphi _0$ for the case of bearing without wear. However, for the case of worn bearing, $\varphi _0$ is calculated iteratively using Eq. (26)^22^.

Figure 2b shows the relationship between $\varphi _0$ and $\epsilon _0$ for worn bearing. The variation of $\varphi _0$ is demonstrated as a function of $\epsilon _0$ and $\delta$. It is observed that, for a constant value of $\epsilon _0$ , $\varphi _0$ decreases as $\delta$ increases. For example, $\epsilon _0$ is set constant at 0.01 and $\delta$ is varied from 0 to 0.5. When $\delta$ = 0, $\varphi _0$ = 1.558 and when $\delta$= 0.5, $\varphi _0$ = 1.111, giving a percentage difference of 28.72% between a non-worn and worn bearing. Moreover, the percentage difference between a non-worn and worn bearing also increases as $\epsilon _0$ increases^24^. For example,$\epsilon _0$ is set constant at 0.7 and $\delta$ is varied from 0 to 0.5. When $\delta$ = 0, $\varphi _0$ = 0.676 and when $\delta$ = 0.5, $\varphi _0$ = 0.007, calculating a percentage difference of 98.95% between a non-worn and worn bearing. The static equilibrium position from the present work in the condition when there is no wear ($\delta = 0$) matches closely to those that were presented by Jamil et al.^20^.

**Figure 3:**
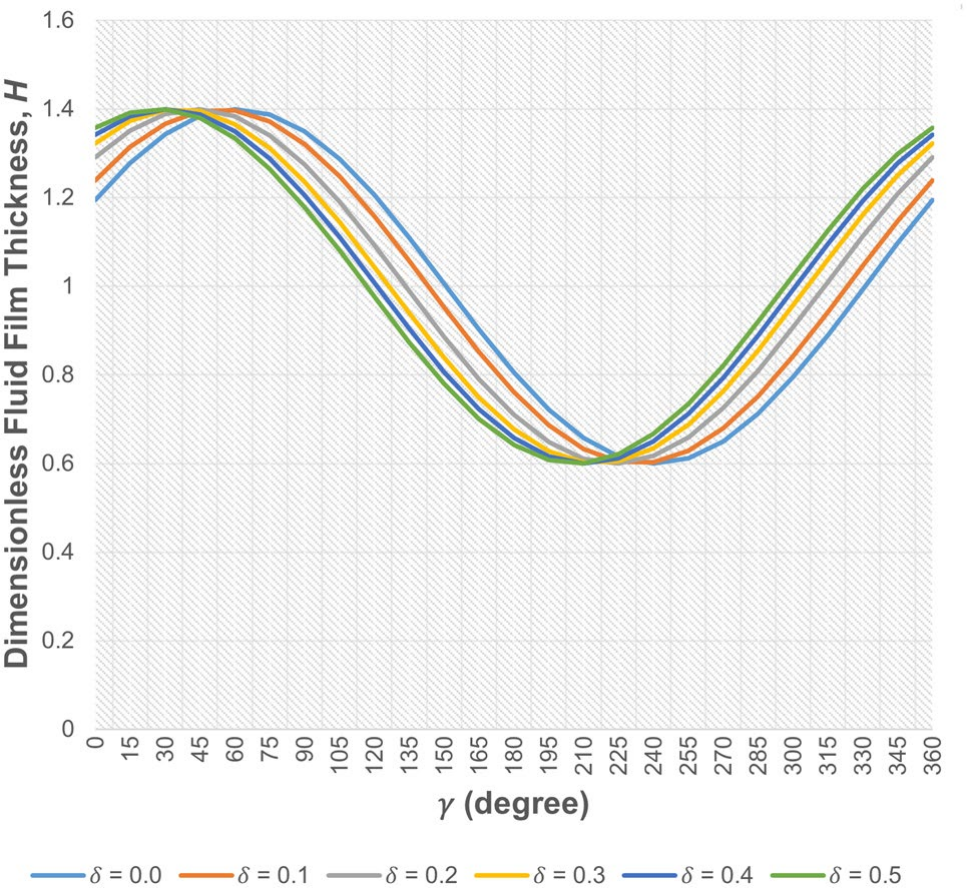
Fluid film thickness profile for the case of $\epsilon _0$ = 0.4 for various wear depth values.

The fluid film thickness in the journal bearing, given in Eqs. (1) and (9), respectively for the non-worn and worn journal bearing, is a function of the attitude angle $\varphi _0$. For a given value of $\epsilon _0$, the attitude angle varies with the depth of wear, as illustrated in Fig. 2b. Therefore, the angular position ($\gamma$) where the minimum film thickness occurs changes with the depth of wear. The fluid film thickness profile for the case of $\epsilon _0$=0.4 is shown in Fig. 3 for various wear depth values. It is observed that as the wear depth increases, the angular position ($\gamma$) where the minimum film thickness occurs decreases.

### Effect of wear on the dynamic coefficients of journal bearings

**Figure 4:**
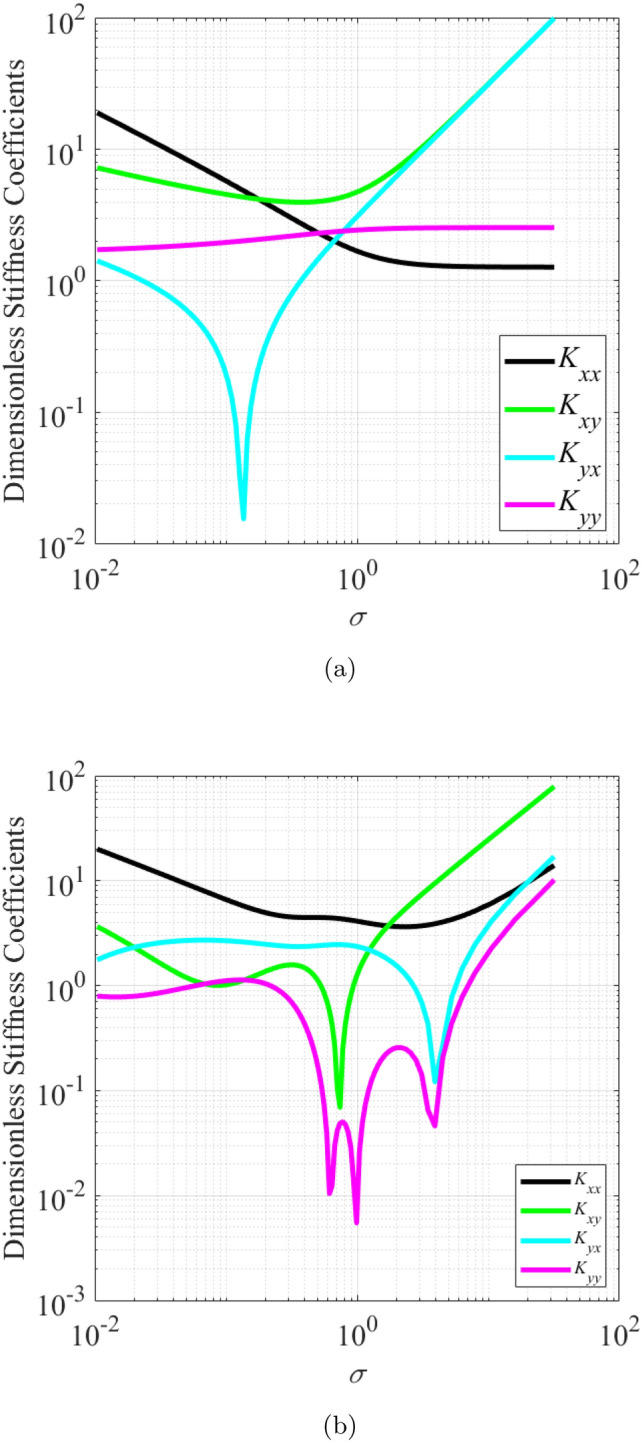
Dimensionless stiffness coefficient as a function of modified Sommerfeld number,$\sigma$, for (**a**) bearing with $\delta = 0$ (**b**) bearing with $\delta = 0.5$.

**Figure 5:**
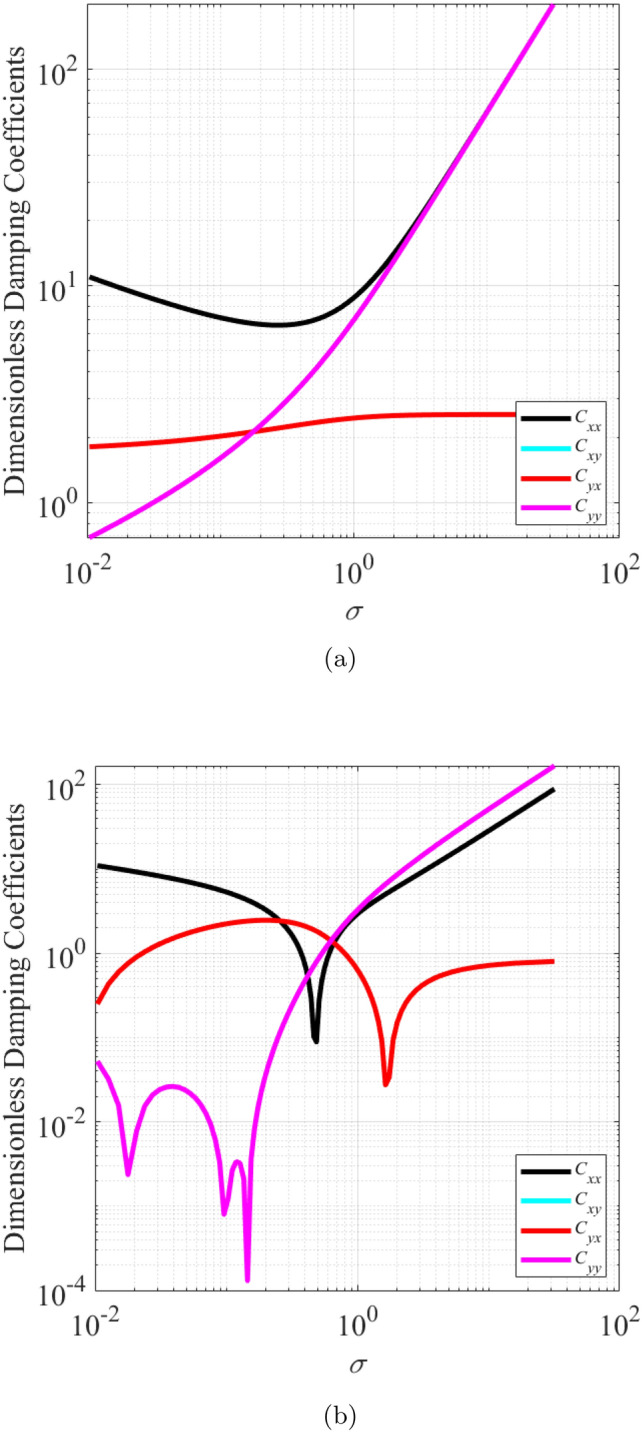
Dimensionless damping coefficient as a function of modified Sommerfeld number,$\sigma$, for (**a**) bearing with $\delta = 0$ (**b**) bearing with $\delta = 0.5$.

The effect of wear on the dynamic coefficients of journal bearings was investigated for values of $\epsilon _0$ between 0.1 and 0.75. The wear depth parameter ratio $\delta$ was varied from 0 to 0.5. Figures 4 and 5 show the dimensionless stiffness and damping coefficient as a function of $\sigma$ for (a) bearing with $\delta$ = 0 (b) bearing with $\delta$ = 0.5. It is observed that wear alters the relation between the direct and the cross coupled stiffness coefficients in both the *X*-direction and the *Y*-direction, especially at low $\sigma$. As reported by Machado and Cavalca^19^, the wear increases the anisotropic characteristics of these coefficients. The dimensionless linearized dynamic coefficient from the present work in the condition when there is no wear ($\delta = 0$) closely matches those that were presented by Jamil et al.^20^.

**Figure 6:**
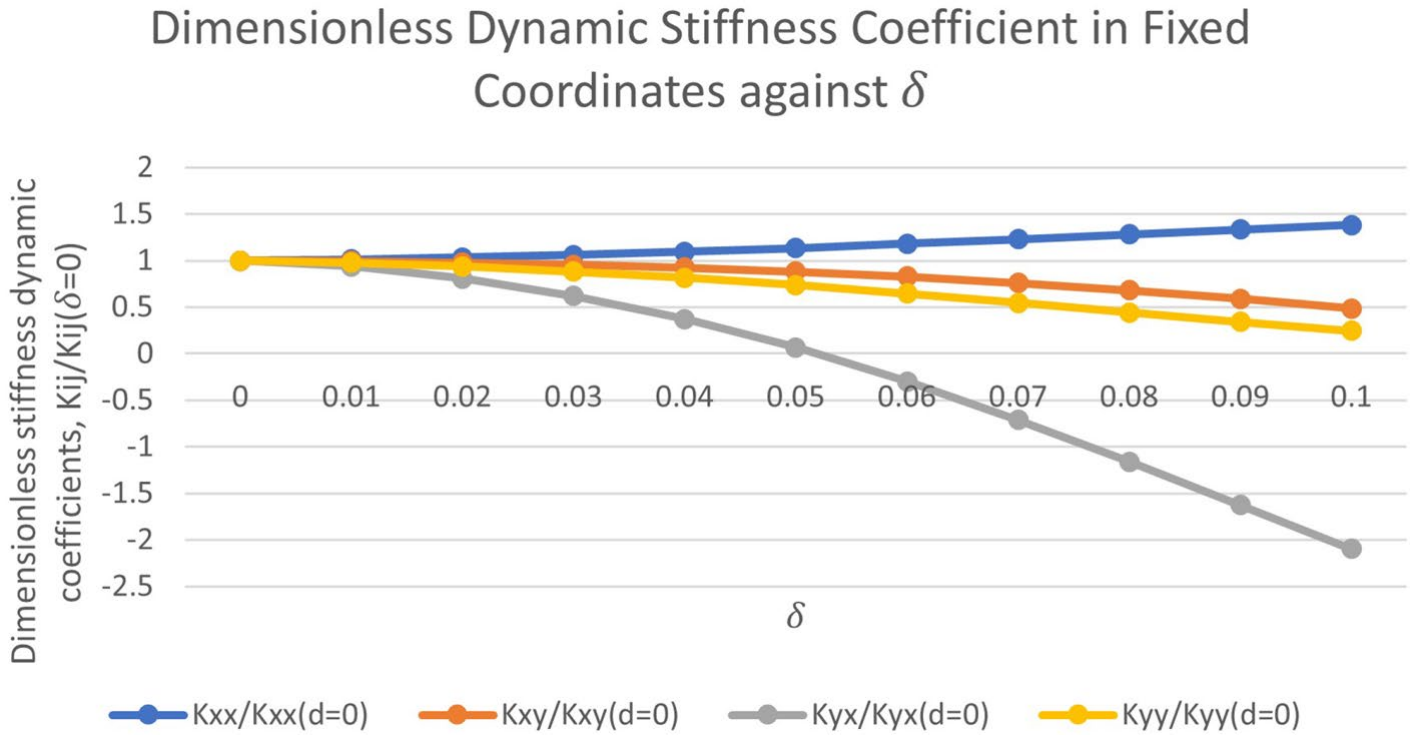
Dimensionless dynamic stiffness coefficient in fixed coordinates against $\delta$ ranging from $\delta = 0$ to $\delta = 0.1$.

Figure 6 shows the dimensionless dynamic stiffness coefficient against $\delta$ ranging from $\delta$ = 0 to $\delta$ = 0.5 when the rotor is operated under high operating regime ($\epsilon _0$ = 0.61). It is observed that $K_{xx}$ increases by 38.37%, $K_{xy}$ reduces by 51.25%, $K_{yx}$ reduces by 309.72%, and $K_{yy}$ reduces by 75.46%.

Dynamic direct stiffness coefficient relates the change in force in one direction to a displacement in the same direction as shown in Eqs. (34) and (35)^25^:

34 $$\begin{aligned} K_{xx}= & {} \bigtriangleup {F_x}/{\bigtriangleup {x}} \end{aligned}$$

35 $$\begin{aligned} K_{yy}= & {} \bigtriangleup {F_y}/{\bigtriangleup {y}} \end{aligned}$$

The nature of the dynamic direct stiffness is to provide a restoring force that pushes the journal back toward its steady-state equilibrium position^25^. In Fig. 6, it is seen that the dynamic direct stiffness coefficients $K_{yy}$ decreases with increasing $\delta$ whereas the direct stiffness coefficient $K_{xx}$ increases with increasing $\delta$. This is because when $\delta$ increases, $\varphi _0$ decreases and $\epsilon _0$ increases which causes the rotor to move closer to the *X*-axis and further in the *X*-direction. As such, the displacement in the *X*-direction, $\bigtriangleup {x}$ and the restoring force in the *X*-direction, $\bigtriangleup {F_x}$, increases whereas the displacement in the *Y*-direction, $\bigtriangleup {y}$, and the restoring force in the *Y*-direction, $\bigtriangleup {F_y}$, reduces. As a result, an increase in $K_{xx}$ and a drop in $K_{yy}$ is observed.

The cross-coupled stiffness relates to a displacement resulted in force component perpendicular to this displacement as given in Eqs. (36) and (37)^25^:

36 $$\begin{aligned} K_{xy}= & {} \bigtriangleup {F_x}/{\bigtriangleup {y}} \end{aligned}$$

37 $$\begin{aligned} K_{yx}= & {} \bigtriangleup {F_y}/{\bigtriangleup {x}} \end{aligned}$$

A net force is produced from the reaction forces resulting from the combination of cross coupled stiffness terms. The net force is tangential to the shaft orbit and follows the direction of the journal’s instantaneous motion. As to produce a forward destabilizing force, the $K_{xy}$ term must be positive and the $K_{yx}$ term negative as shown in Fig. 6 for values of $\delta >0.05$. As a result, energy is added to the system and the rotor-bearing system is destabilized by the resultant force from the cross-coupling terms^26^.

The shaft motion within the bearing is usually demonstrated by an elliptical orbit. The area enclosed by the elliptical whirl orbit in the $X-Y$ plane is represented by the following expression^26^:

38 $$\begin{aligned} A=\pi \bar{x}\bar{y}sin({\phi _x - \phi _y}) \end{aligned}$$

where,

$\bar{x}$ is the absolute magnitude of rotor vibration response in the *X*-direction.

$\bar{y}$ is the absolute magnitude of rotor vibration response in the *Y*-direction.

$\phi _x$ is the phase angle of vibration response in the *X*-direction measured counter-clockwise from the *X*-axis.

$\phi _y$ is the phase angle of vibration response in the *Y*-direction measured counter-clockwise from the *Y*-axis.

It is seen that the area can be a positive or negative number with positive being for counter-clockwise whirling and negative for clockwise whirling. The energy input, *E*, produced by the bearing coefficients is calculated by integrating the multiplication product of force and displacement around the closed curve of the ellipse. For the cross coupled stiffness coefficients, Zeidan et al.^27^ has shown that:

39 $$\begin{aligned} E = A(K_{xy} - K_{yx}) \end{aligned}$$

From Eq. (39), it can be noted that the cross-coupling’s destabilizing effect is directly proportional to the area of the whirl orbit and with the net difference between $K_{xy}$ and $K_{yx}$. It is also noted that *E* = 0, if $K_{xy}$ and $K_{yx}$ are equal and have identical sign. The worst combination is when $K_{xy}$ and $K_{yx}$ are equal and opposite in sign which produces a circular orbit^27^. Rotor systems are unique in that $K_{xy} \ne K_{yx}$ and usually $K_{xy} > 0, K_{yx} < 0$^25^. It can be observed in Fig. 6 that $K_{xy}\ne K_{yx}$ and $K_{xy}>0$ for values of $\delta$=0 to $\delta$=0.1. It is also noted that, when $\delta \le 0.05$, $K_{xy}$ and $K_{yx}$ have the same positive sign and when $\delta >0.05$, $K_{xy}$ remain positive whereas $K_{yx}$ became negative which makes the net difference between $K_{xy}$ and $K_{yx}$ larger for $\delta >0.05$ as compared to when $\delta \le 0.05$. A larger net difference between $K_{xy}$ and $K_{yx}$ contributes to a larger energy input as per Eq. (39). Also, when the signs are the same, the cross-coupling has the effect of increasing the apparent asymmetry in $K_{xx}$ and $K_{yy}$^26^. Thus, it can be deduced that when $\delta \le 0.05$ the apparent asymmetry in $K_{xx}$ and $K_{yy}$ increases (rotor-bearing system becomes more stable) whereas when $\delta >0.05$ the apparent asymmetry in $K_{xx}$ and $K_{yy}$ decreases (rotor-bearing system becomes more unstable).

He et al.^25^ mentioned that large asymmetry of $K_{xx}$ and $K_{yy}$ is the main cause of split critical speeds and non-circular (elliptical) orbit shapes. Incorporating asymmetry into the direct stiffnesses has the well-known effect of producing flat, elongated whirl orbits in the vertical and horizontal direction. This reduces the area of the orbit and thereby lessens the energy input by cross-coupled stiffness. Sufficient asymmetry flattens the orbit completely to a straight line, and the unstable system is rendered stable as the energy input becomes zero^26^.

**Figure 7:**
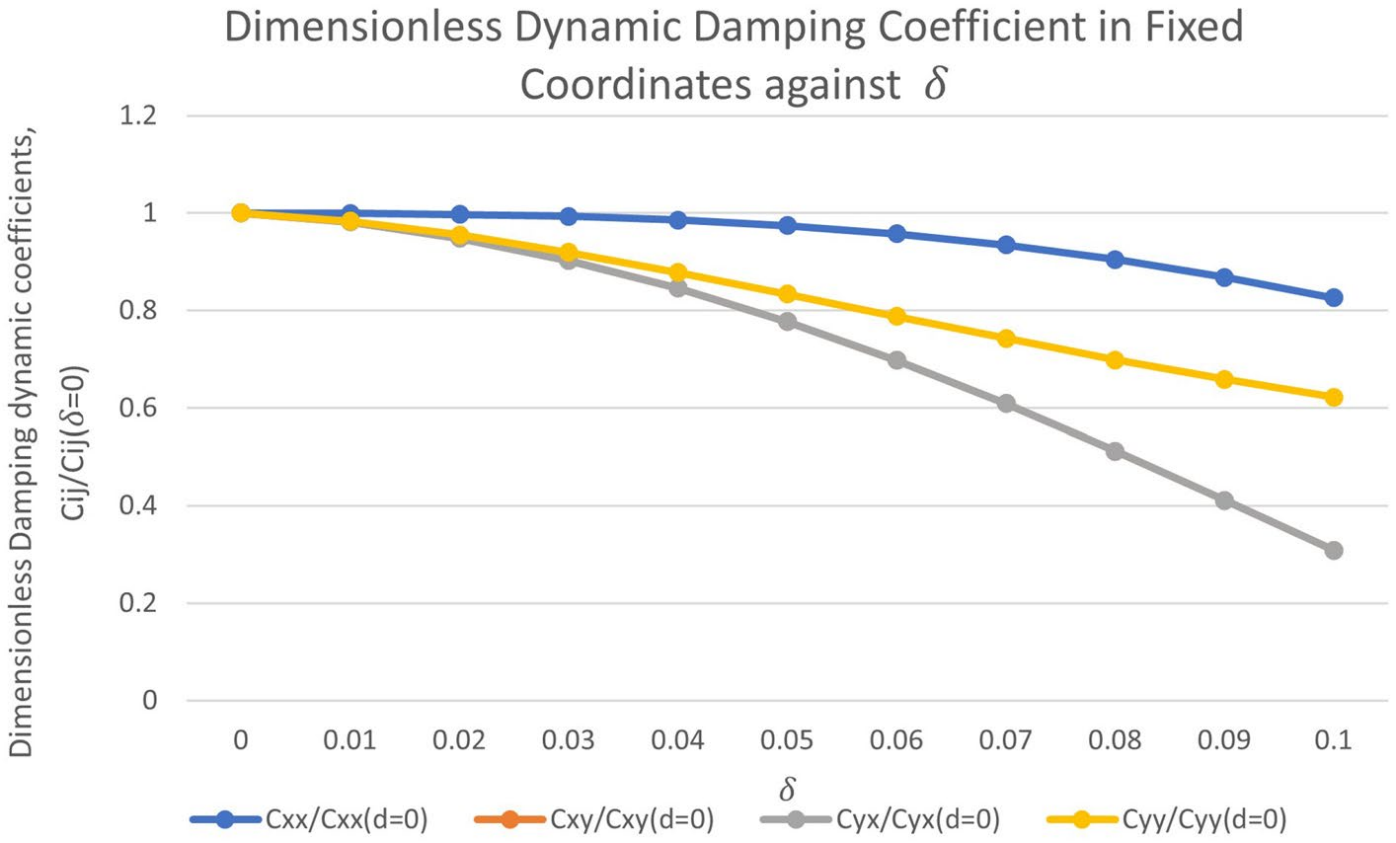
Dimensionless dynamic damping coefficient in fixed coordinates against $\delta$ ranging from $\delta = 0$ to $\delta = 0.1$.

Figure 7 shows the dimensionless damping dynamic coefficient against $\delta$ for values of $\delta = 0$ to $\delta = 0.5$ when the rotor is operated under high operating regime ($\epsilon = 0.61$). It is observed that $C_{xx}$ decreases by 17.38 %, $C_{xy}$ decreases by 69.10 %, $C_{yx}$ decreases by 69.10 %, and $C_{yy}$ decreases by 37.78 %.

Damping, which is generally assumed to be positive, dissipates energy from the dynamic motion of the rotor-bearing system and thus promotes stability. The direct damping coefficient is related to the change in force due to a small change in velocity as shown in Eqs. (40) and (41)^25^.

40 $$\begin{aligned} C_{xx}= & {} \bigtriangleup {F_x}/{\bigtriangleup {\dot{x}}} \end{aligned}$$

41 $$\begin{aligned} C_{yy}= & {} \bigtriangleup {F_y}/{\bigtriangleup {\dot{y}}} \end{aligned}$$

Due to the rotor’s whirling motion, the combination of the two direct damping coefficients, $C_{xx}$ and $C_{yy}$ produces a force that is tangential to the vibration orbit. This direct damping force acts against the whirling motion, helping to retard or slow it^25^. As for cross-coupled damping, it is often found that the effect of cross-coupled stiffness outweighs the effect of cross-coupled damping. This is because the resulting energy from cross-coupled damping is identically zero. Hence, in the absence of cross-coupled stiffness, cross-coupled damping cannot drive the rotor-bearing system unstable by itself^26^. It can be concluded that in general, the effect of wear on the dynamic coefficients of journal bearings is such that the dynamic coefficients except for $K_{xx}$ decrease with increasing $\delta$. This result is consistent with the work of Zeidan & Herbage^27^.

### Effect of wear on the vibration response of the rotor

The numerical simulation to study the effect of wear on the vibration response of the rotor was undertaken for 3 different loaded operating regime namely low loaded operating regime ($0.1 \le \epsilon _0 \le 0.2$), moderately loaded operating regime ($0.3 \le \epsilon _0 \le 0.5$) and highly loaded operating regime ($0.6 \le \epsilon _0 \le 0.75$)^28^. The unbalance parameter *u* was set at 0.05. The dimensionless journal mass *m* was varied from 2 to 73. The wear depth parameter ratio $\delta$ was varied from 0 to 0.5 and the results are presented for $\delta$ increments of 0.1.

#### Low loaded operating regime ($0.1 \le \epsilon _0 \le 0.2$)

**Figure 8:**
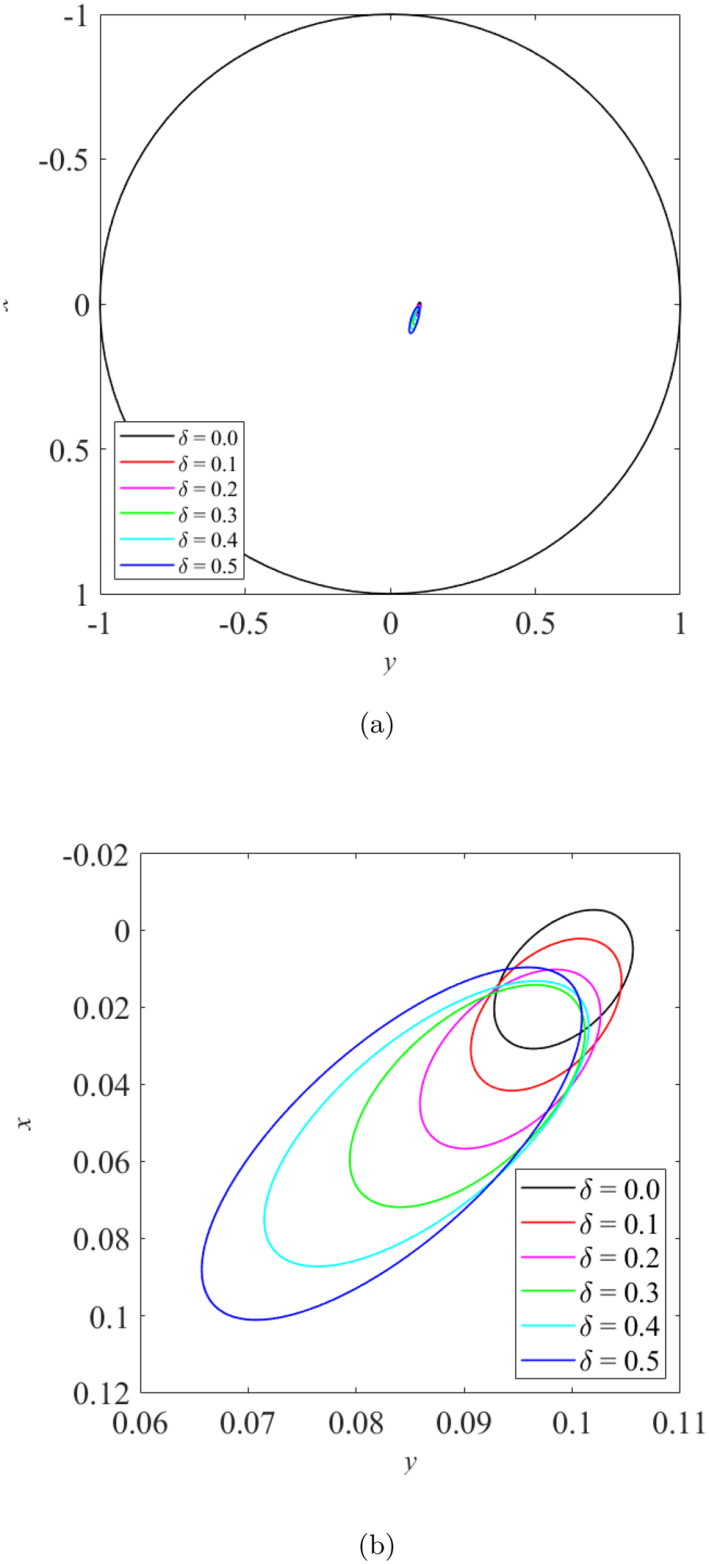
Rotor whirl orbit for low loaded operating regime ($\epsilon _0 = 0.1$) (**a**) with a boundary view of unit circle (**b**) enlarged view.

**Figure 9:**
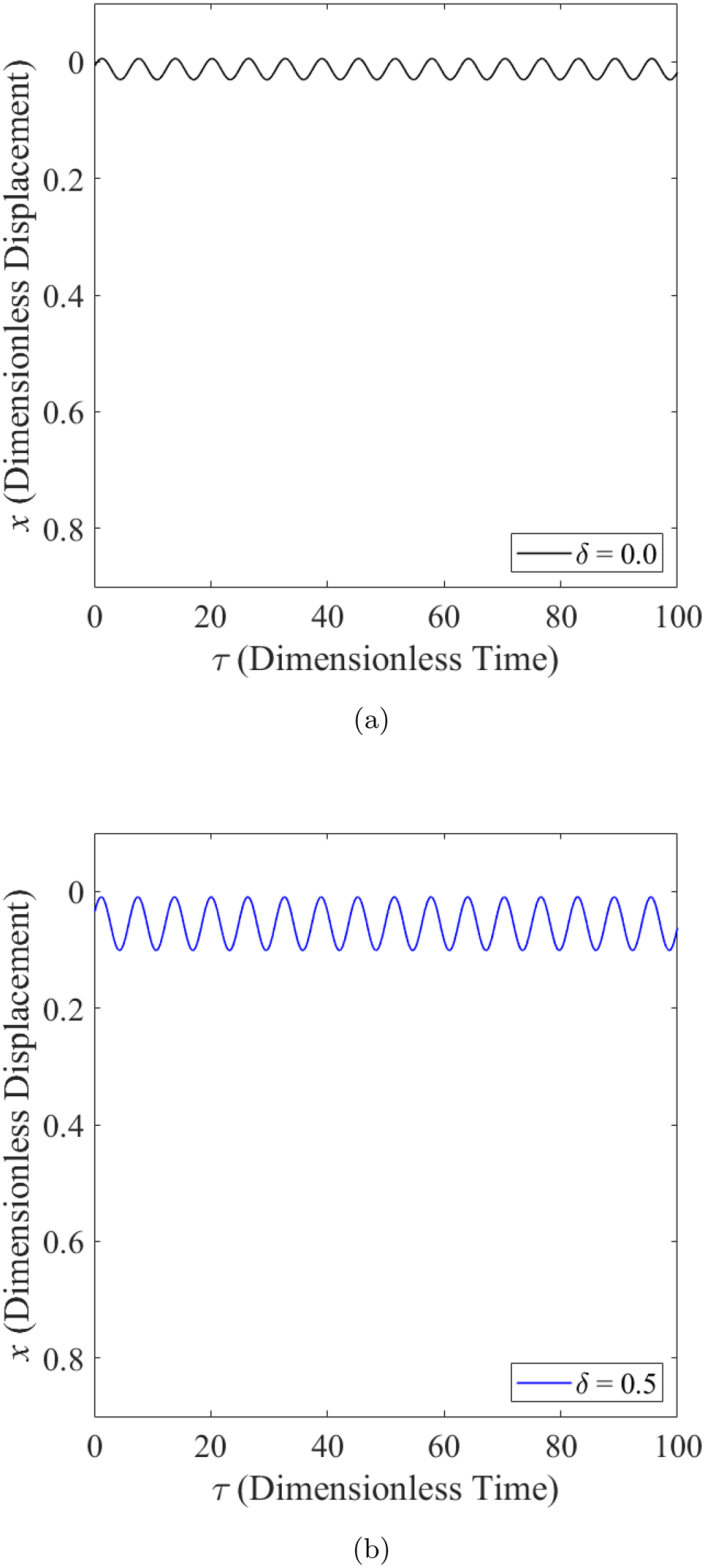
Vibration response in the *X*-direction for low loaded operating regime ($\epsilon _0 = 0.1$) for (**a**) $\delta = 0$ (**b**) $\delta = 0.5$.

**Figure 10:**
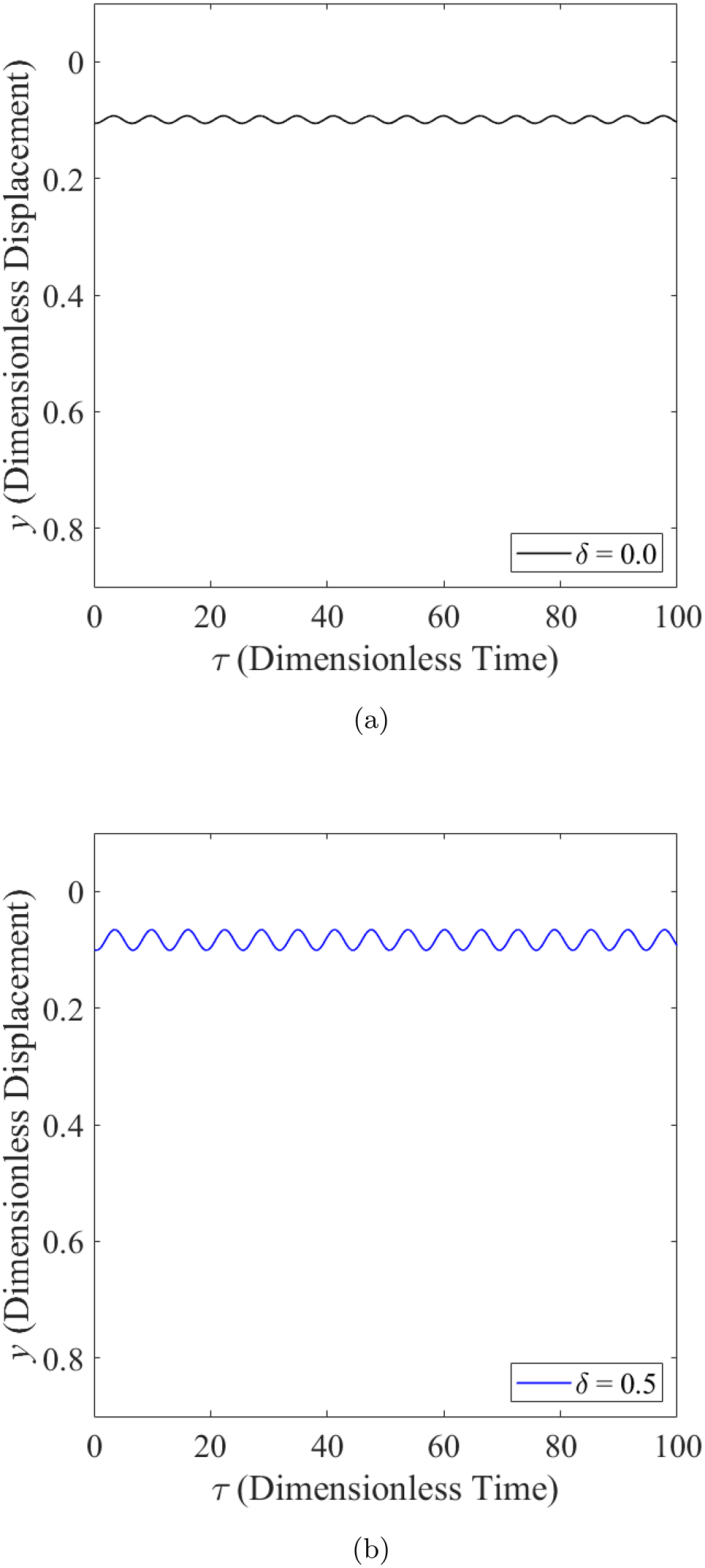
Vibration response in the *Y*-direction for low loaded operating regime ($\epsilon _0 = 0.1$) for (**a**) $\delta = 0$ (**b**) $\delta = 0.5$.

The effect of wear on the vibration response of rotor when the rotor is operated under low loaded operating regime ($\epsilon _0 = 0.1$) whereby $m=2$ is shown from Fig. 8 to Fig. 10. Figure 8 shows the rotor whirl orbit for low loaded operating regime ($\epsilon _0 = 0.1$); (a) with a boundary view of unit circle (b) enlarged view. Figure 8a and b show that the rotor whirl orbit increases in size with increasing $\delta$. Figure 9 shows the vibration response in the *X*-direction for low loaded operating regime ($\epsilon _0 = 0.1$) for (a) $\delta = 0$ (b) $\delta = 0.5$. The magnitude of *X* response has increased from 0.018 in Fig. 9a to 0.046 in Fig. 9b which yields a net increase of 161% when $\delta$ increases from $\delta = 0$ to $\delta = 0.5$. Figure 10 shows the vibration response in the Y-direction for low loaded operating regime ($\epsilon _0 = 0.1$) for (a) $\delta = 0$ (b) $\delta = 0.5$. The magnitude of *Y* has increased from 0.006 in Fig. 10a to 0.018 in Fig. 10b which yields a net increase of 179% when $\delta$ increases from $\delta = 0$ to $\delta = 0.5$.

#### Moderately loaded operating regime ($0.3 \le \epsilon _0 \le 0.5$)

**Figure 11:**
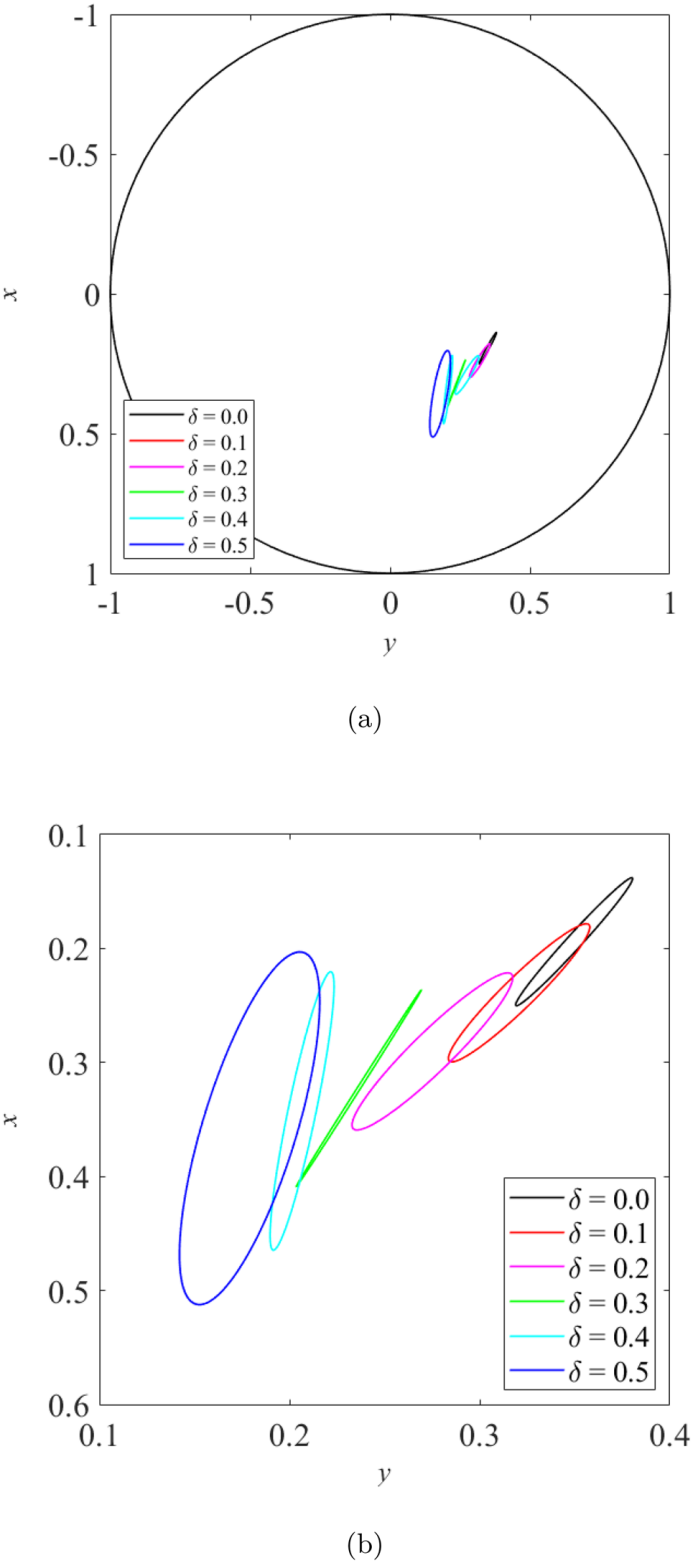
Rotor whirl orbit for moderately loaded operating regime ($\epsilon _0 = 0.4$) (**a**) with a boundary view of unit circle (**b**) enlarged view.

**Figure 12:**
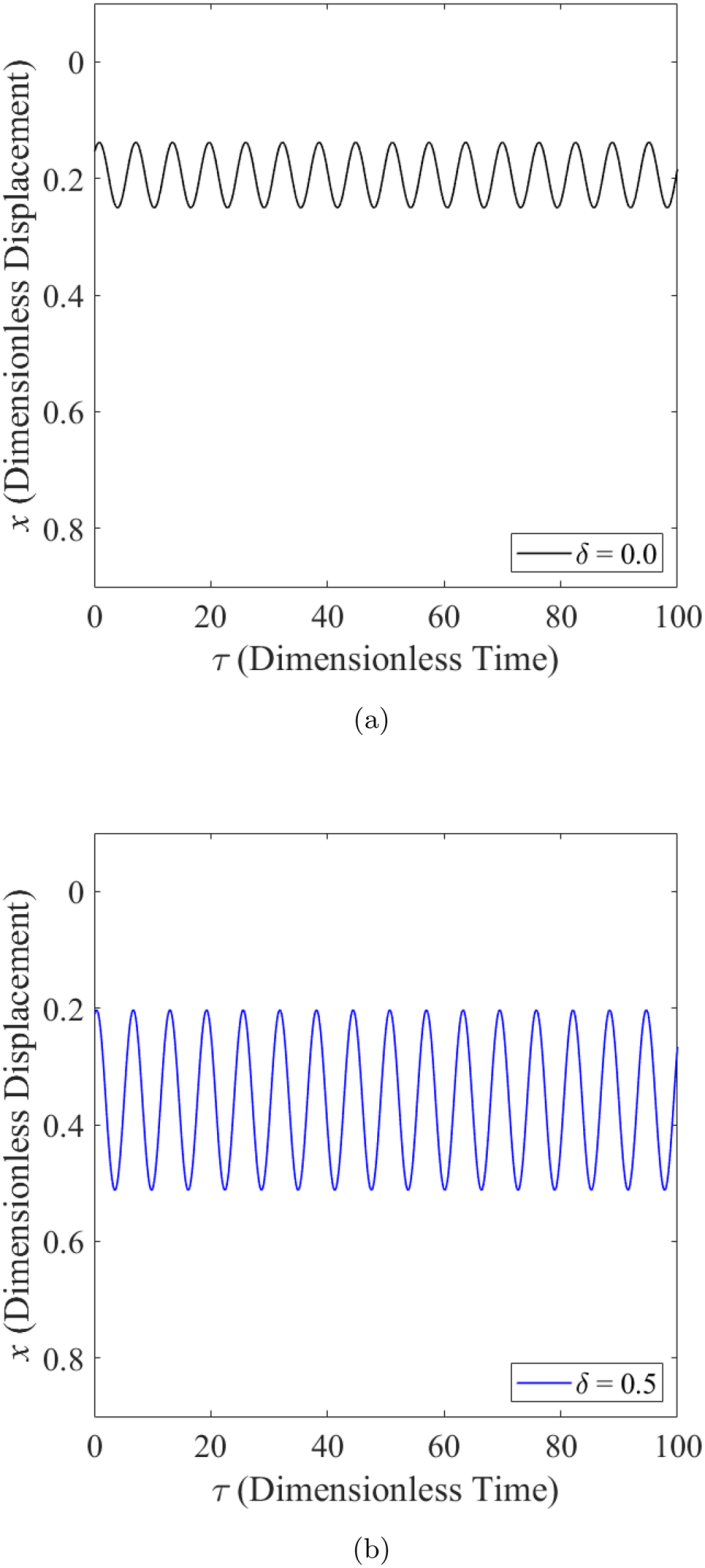
Vibration response in the *X*-direction for moderately loaded operating regime ($\epsilon _0 = 0.4$) for (**a**) $\delta = 0$ (**b**) $\delta = 0.5$.

**Figure 13:**
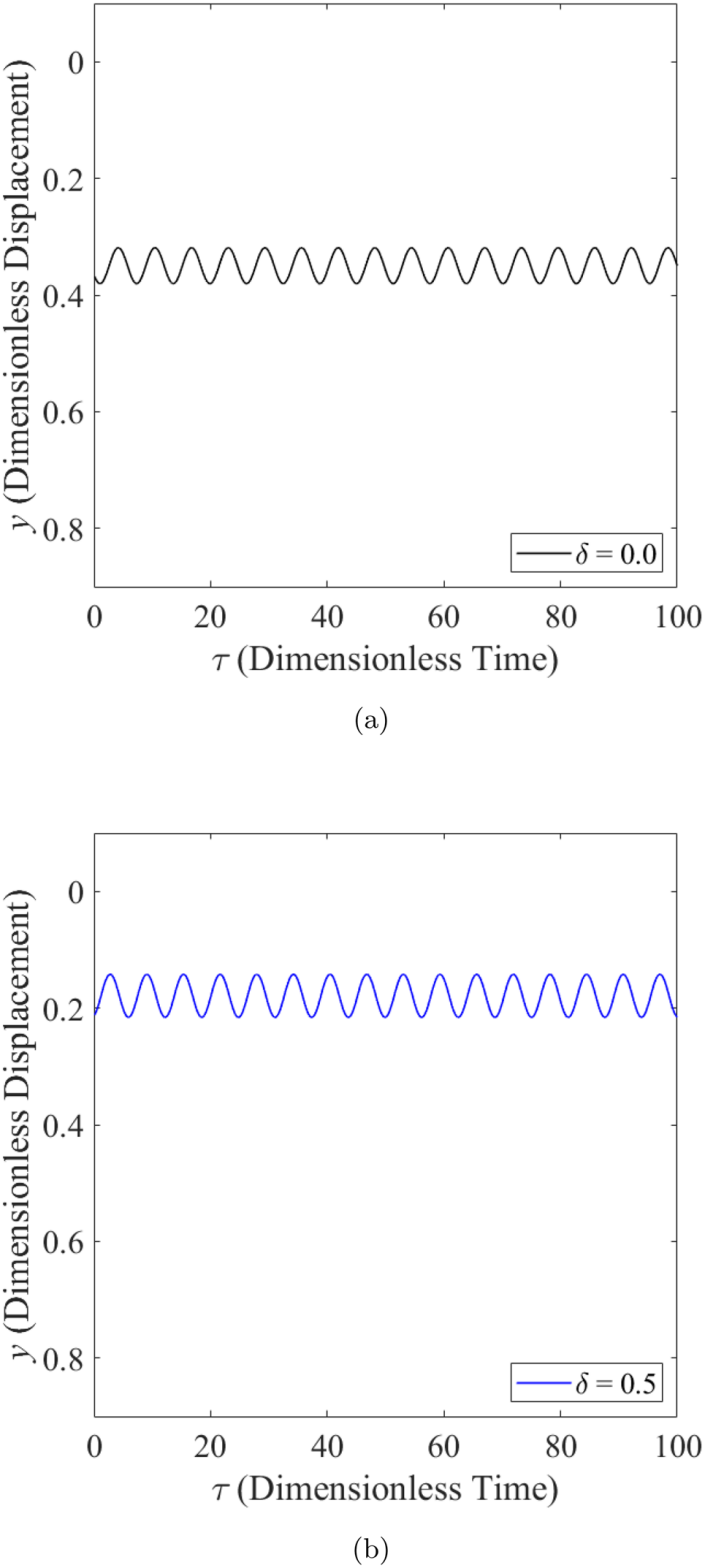
Vibration response in the *Y*-direction for moderately loaded operating regime ($\epsilon _0 = 0.4$) for (**a**) $\delta = 0$ (**b**) $\delta = 0.5$.

When the rotor is operated under moderately loaded operating regime ($\epsilon _0= 0.4$) whereby $m=12.5$, the effect of wear on the vibration response of rotor can be seen in Figs. 11, 12 and 13. Figure 11 shows the rotor whirl orbit for moderately loaded operating regime ($\epsilon _0 = 0.4$); (a) with a boundary view of unit circle (b) enlarged view. Figure 11a and b show that the rotor whirl orbit increases in size with increasing $\delta$. Figure 12 shows the vibration response in the *X*-direction for moderately loaded operating regime ($\epsilon _0 = 0.4$) for (a) $\delta = 0$ (b) $\delta = 0.5$. The magnitude of *X* response increased from 0.056 in Fig. 12a to 0.154 in Fig. 12b, which gives a net increase of 176% when $\delta$ increases from $\delta = 0$ to $\delta = 0.5$. Figure 13 shows the vibration response in the *Y*-direction for moderately loaded operating regime ($\epsilon _0 = 0.4$) for (a) $\delta = 0$(b) $\delta = 0.5$. The magnitude of *Y* response increased from 0.031 in Fig. 13a to 0.037 in Fig. 13b which yields a net increase of 20.4% when $\delta$ increases from $\delta = 0$ to $\delta = 0.5$.

#### Highly loaded operating regime ($0.6 \le \epsilon _0 \le 0.75$)

**Figure 14:**
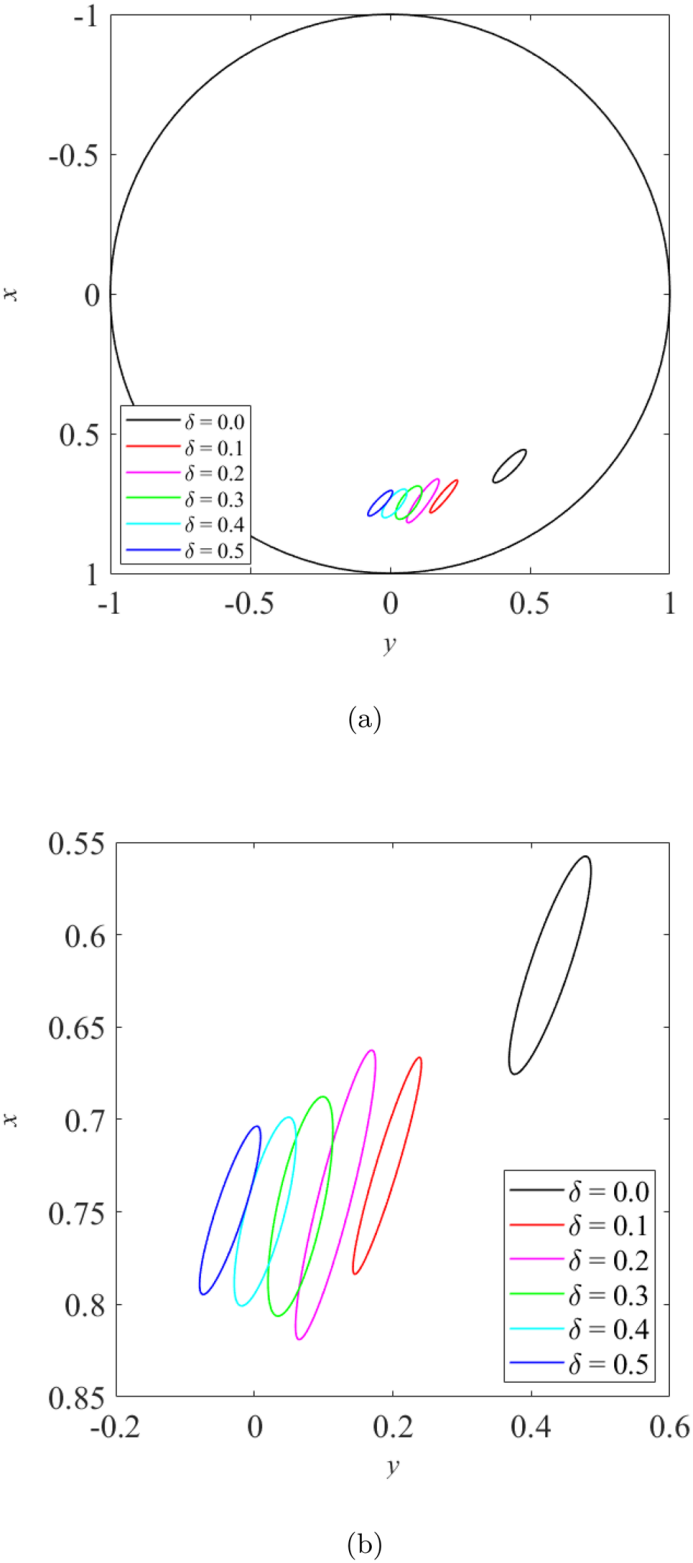
Rotor whirl orbit for highly loaded operating regime ($\epsilon _0 = 0.75$) (**a**) with a boundary view of unit circle (**b**) enlarged view.

**Figure 15:**
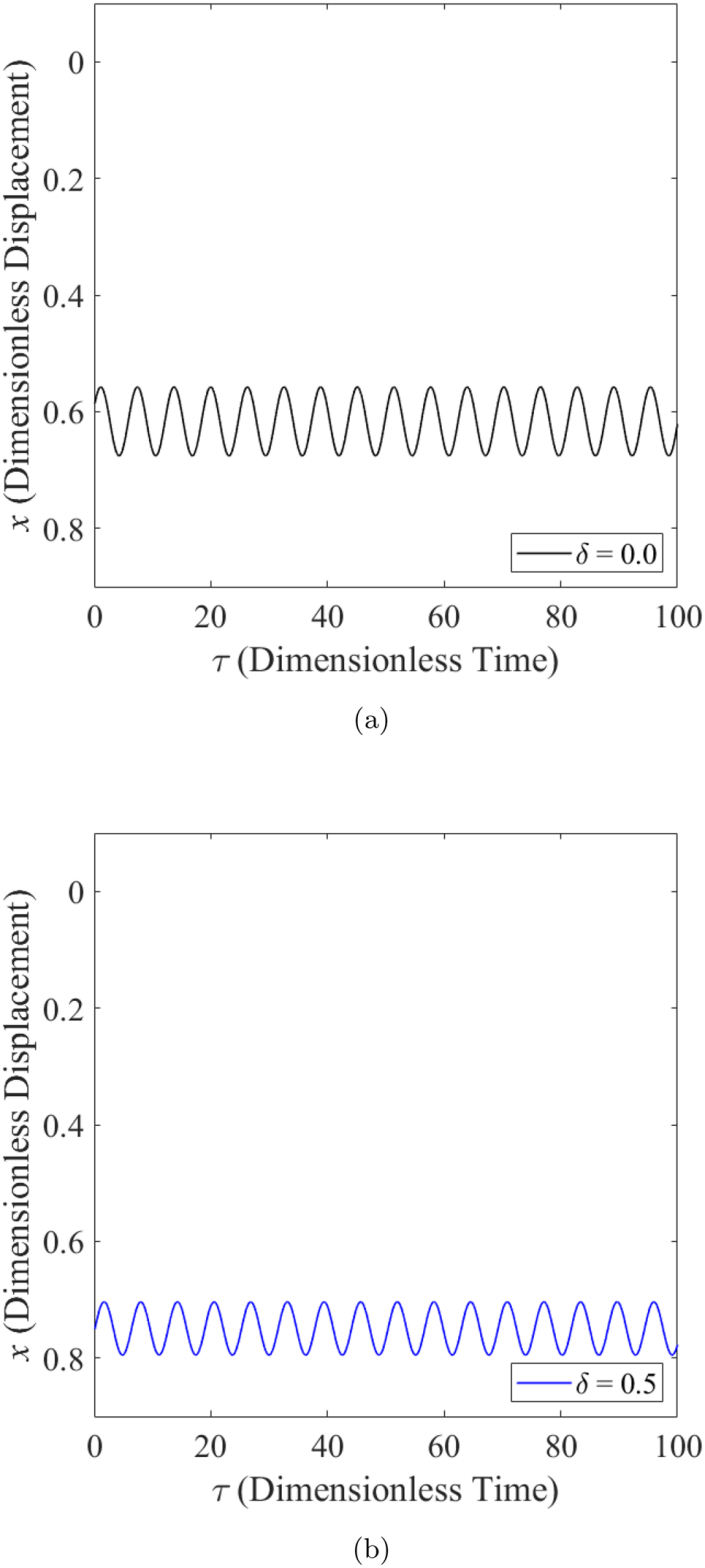
Vibration response in the *X*-direction for highly loaded operating regime ($\epsilon _0 = 0.75$) for (**a**) $\delta = 0$ (**b**) $\delta = 0.5$.

**Figure 16:**
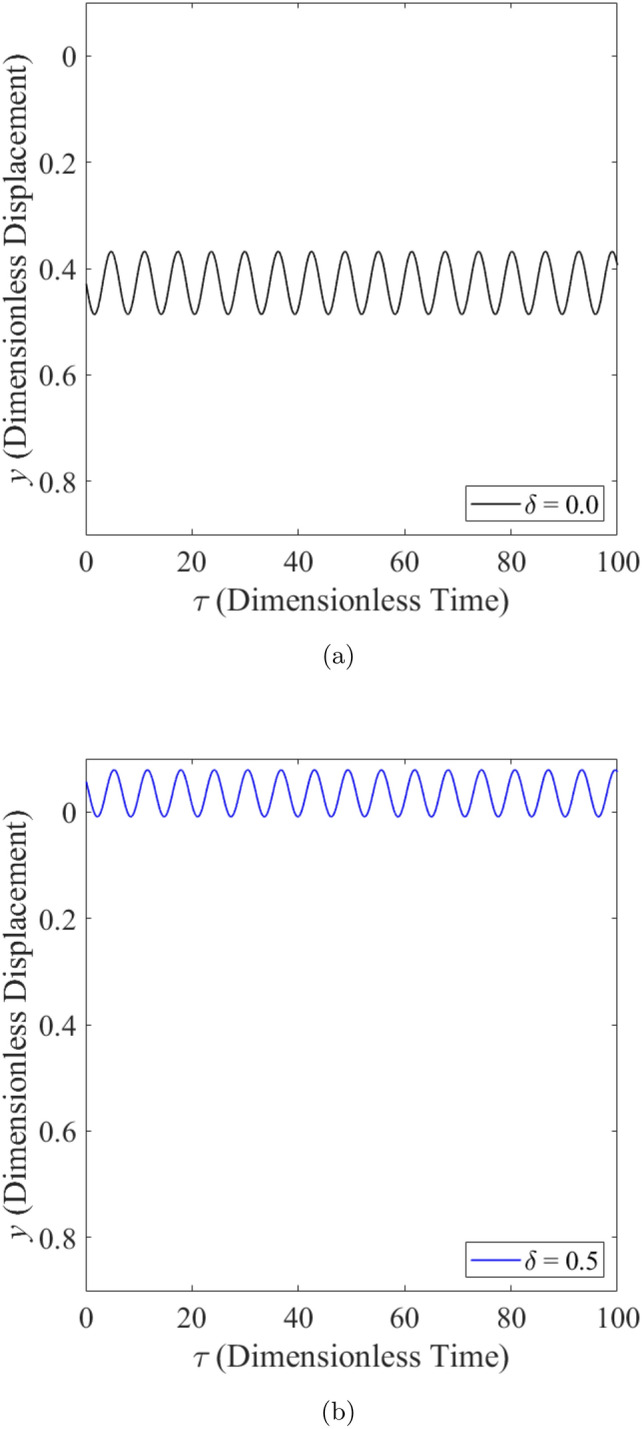
Vibration response in the *Y*-direction for highly loaded operating regime ($\epsilon _0 = 0.75$) for (**a**) $\delta = 0$ (**b**) $\delta = 0.5$.

The effect of wear on the vibration response of rotor when the rotor is operated under highly loaded operating regime ($\epsilon _0 = 0.75$) whereby $m=73$ is observed in Figs. 14, 15 and 16. Figure 14 shows the rotor whirl orbit for highly loaded operating regime ($\epsilon _0=0.75$); (a) with a boundary view of unit circle (b) enlarged view. Figure 14a and b show that the rotor whirl orbit increases in size with increasing $\delta$. Figure 15 shows the vibration response in the *X*-direction for highly loaded operating regime ($\epsilon _0 = 0.75$) for (a) $\delta = 0$ (b) $\delta = 0.5$. The magnitude of *X* response increased from 0.059 in Fig. 15a to 0.443 in Fig. 15b which yields a net increase of 650.8% when $\delta$ increases from $\delta = 0$ to $\delta = 0.5$. Figure 16 shows the vibration response in the *Y*-direction for highly loaded operating regime ($\epsilon _0 = 0.75$) for (a) $\delta = 0$ (b) $\delta = 0.5$. The magnitude of *Y* response dropped from 0.059 in Fig. 16a to 0.035 in Fig. 16b which yields a net drop of 41.0% when $\delta$ increases from $\delta = 0$ to $\delta = 0.5$.

In general, the effect of wear on the vibration response of the rotor is such that when $\delta$ increases, the vibration response of the rotor in the *X*-and *Y*-direction under all 3 loaded operating regime increases with the exception of the response in the *Y*-direction for the highly loaded operating regime. For a constant value of $\epsilon _0$ , $\varphi _0$ decreases as $\delta$ increases from $\delta = 0$ to $\delta = 0.5$. This means that the rotor is moving closer to the vertical axis and when $\delta$ increases, the fluid-film thickness increases. As the fluid-film thickness increases, the dynamic stiffness reduces and when subjected to the same unbalance force, the amplitude of the rotor’s displacement increases. However, the vibration response of the rotor in *Y*-direction when the rotor is operating under highly loaded operating regime decreases with increasing $\delta$. This is because as $\delta$ increases, the clearance and fluid-film thickness in the *Y*-direction reduces. As such, the dynamic stiffness in that direction increases and when subjected to the same unbalance force, the amplitude of the rotor’s displacement reduces as $\delta$ is increased from 0 to 0.5.

## Conclusion

The effect of wear in journal bearings on the behavior of a rigid rotor, particularly on the static equilibrium position, the dynamic coefficients, and the vibration response of the rotor was investigated. The effect of wear on the static equilibrium position of the rotor was examined for values of $\epsilon _0$ between 0.1 and 0.9 with $\delta$ varied from 0 to 0.5, in increments of 0.1. The results showed that $\epsilon _0$ has an inverse relationship with $\varphi _0$, where increase in the value of $\epsilon _0$ cause the value of $\varphi _0$ to decrease. The effect of wear on the dynamic coefficients of journal bearings was investigated for values of $\epsilon _0$ between 0.1 and 0.75 with $\delta$ varied from 0 to 0.5. The numerical results showed that the dynamic coefficients, except for $K_{xx}$, decrease with increasing $\delta$. The effect of wear on the vibration response of a rigid rotor was undertaken for three different operating regimes of the journal bearings, namely low loaded operating regime, moderately loaded operating regime and highly loaded operating regime. The unbalance parameter *u* was set at 0.05. The dimensionless journal mass *m* was varied from 2 to 73. $\delta$ was varied from 0 to 0.5 in increments of 0.1. The numerical results showed that increasing the wear depth in the journal bearings generally caused an increase in the vibration response amplitude of the rigid rotor in the *X*-and *Y*-directions, for all three cases of operating regimes, with the exception of the response amplitude of the rotor in the *Y*-direction for the highly loaded operating regime.

## Data Availability

The datasets generated and/or analyzed during the current study are available from the corresponding author upon reasonable request.

## Code availability

The code generated during the current study are available from the corresponding author upon reasonable request.
